# The positive aspects of caregiving in dementia: A scoping review and bibliometric analysis

**DOI:** 10.3389/fpubh.2022.985391

**Published:** 2022-09-14

**Authors:** Jun Wang, Xuelian Li, Weichu Liu, Bing Yang, Qinghua Zhao, Yang Lü, Mingzhao Xiao

**Affiliations:** ^1^Department of Urology, The First Affiliated Hospital of Chongqing Medical University, Chongqing, China; ^2^Department of Gynecology, The First Affiliated Hospital of Chongqing Medical University, Chongqing, China; ^3^Department of Nursing, Stomatological Hospital of Chongqing Medical University, Chongqing, China; ^4^Department of Nursing, The First Affiliated Hospital of Chongqing Medical University, Chongqing, China; ^5^Department of Geriatrics, The First Affiliated Hospital of Chongqing Medical University, Chongqing, China

**Keywords:** dementia, positive aspects of caregiving, scoping review, bibliometric analysis, Alzheimer's disease

## Abstract

**Purpose:**

The increasing incidence of dementia and home-based care exposes family caregivers to a variety of challenges as they endure strong stressors underlying the caregiver role. Despite growing publications on positive aspects of caregiving in dementia, few studies have identified the extent, nature, and gaps in the existing literature based on a holistic view. The aim was to identify key issues and a holistic view of literature regarding positive aspects of caregiving in dementia.

**Methods:**

A scoping review was conducted underlying a five-stage framework by Arksey and O'Malley. Five databases, including PubMed, CINAHL, PsycINFO, Embase, and Web of Science, were searched, and references were listed accordingly. Data were extracted by two researchers, comprising article characteristics, forms of positive aspects of caregiving and measurements, theories, forms of dementia and family caregiver, and keywords. Descriptive statistics and narrative synthesis were performed to analyze data. Network analysis of keywords and authors was conducted using VOSviewer software. Word cloud analysis of titles was examined by NVivo.

**Results:**

The review included 230 articles, most of which have been published in the last decade (62.61%). Most articles (40.00%) were contributed by the United States. Cross-sectional studies (41.30%) ranked first, followed by qualitative studies (13.48%). Over a quarter of the literature (28.26%) focused on Alzheimer's care, and nearly 90% included all forms of family caregivers. The Positive Aspects of Caregiving Scale and stress coping theory were most frequently cited. Four clusters dominated by Casey D, Quinn C, Joling KJ, and Teahan A were identified in the network of co-authorship. Six themes were identified: current situations of caregiver experiences, antecedents, consequences, measurement development, effects of interventions, and the concept of positive aspects of caregiving. These were in line with network analysis of keywords and word cloud analysis of titles.

**Conclusions:**

Positive aspects of caregiving in dementia have been widely concerned, but most of them are based on the theory of the negative stress process and are limited to current situations and influencing factors. Building theories focus on the positive aspects of caregiving, subsequently developing a comprehensive measurement and effective interventions, should be further studied.

## Introduction

Dementia, listed by WHO as a global public health priority, has attracted worldwide attention. An estimated 50 million people worldwide are currently living with dementia, and the prevalence of dementia is expected to double in the next 30 years (1). Dementia with progressively cognitive deficits and a variety of neuropsychiatric symptoms leads to disability and care dependence, which emerges as huge demands (e.g., costs and human resources) of long-term care, especially, COVID-19 exacerbates the home-care challenges (2–4). A majority of people with dementia reside at home and/or with the community and family members play a vital role in dementia care services (5). Nevertheless, for most family caregivers without professional education on dementia care and management of neuropsychiatric symptoms, the process of caregiving is recognized as a stressful experience with substantial physical, emotional, and financial burdens (6, 7). Extensive research has focused on the negative aspects of caregiving in dementia, and its influencing factors and detrimental impacts on caregivers' life quality and well-being (8–10). Solely focusing on the negative aspects of caregiving has been critiqued as an incomplete picture of caregivers' well-being (11). Meanwhile, emerging evidence suggested that positive aspects of caregiving coexisted in such a stressful process, which offered new horizons for a better understanding of the caregiving experience of dementia (12).

Currently, the concept of positive aspects of caregiving has not reached an agreement regarding its definition, and multiple studies have carried out a synthesis of dimensions based on the literature. Positive aspects are considered for the extent to which the role of the caregiver is inspiring and rewarding, generating positive consequences and enriching individuals' life experiences (13). Satisfaction, rewards, competence, benefit, meaning, personal growth, and a sense of duty are often described as positive aspects in the literature (14, 15). 16 reviewed studies on the conception of positive aspects of caregiving, and they identified four dimensions of positive aspects of caregiving in terms of a sense of personal accomplishment and gratification, feelings of mutuality in a dyadic relationship, an increase of family cohesion and functionality, and a sense of personal growth and purpose in life. Since positive and negative aspects of caregiving are coexistent, instead of separately located in independent ends, ignoring positive aspects of caregiving would limit overall understanding of caregiving adaption, thereby detrimental to the development of interventions. Several studies have reported that the concept of positive aspects of caregiving is a protective factor for caregivers' life quality and well-being (16, 17). Additionally, it could be considered a mediator to reduce negative impacts on health outcomes (18). Therefore, strengthening caregivers' adaptation means going beyond alleviating caregiver burden to enhance positive aspects of caregiving.

In accordance with this, an increasing body of information has been emerged to explore this phenomenon. However, what exactly is being studied in this area of positive aspects of caregiving is not yet clear. A comprehensive systematic review is necessary to be conducted, helping researchers a better understanding of research perspectives, methods, theories, challenges, and trends within international literature on positive aspects of caregiving. Several systematic reviews and/or meta-analyses have been conducted to view specific fields of positive aspects of caregiving, comprising of the following topics: (a) concept analysis (19, 20). (b) positive psychology outcome measurements (21). (c) reviews on particular elements, such as resilience and competence (22–24). (d) impacts of positive aspects of caregiving on well-being (25), and (e) the effects of psychosocial and/or educational interventions on caregiving, especially the concept of positive aspects of caregiving as one of the indicators of outcome evaluation (26, 27). Overall, most of these reviews have primarily explored specific elements of positive aspects of caregiving, and the concept of positive aspects of caregiving is an affiliated indicator, without overall views of literature focused on the positive aspects of caregiving. Thus, a holistic view of comprehensive literature is essential for researchers to grasp future trends of positive aspects of caregiving in dementia.

Scoping review, a popular review method for synthesizing research evidence is suitable for topics that have not been extensively reviewed or for large volumes of literature with complexity or heterogeneity (28, 29). It can draw a map of the extent, nature, and gaps of the existing literature without losing research robustness and rigorous quality assessments, thereby facilitating the dissemination of knowledge (30). According to these descriptions and our research objectives, scoping review is the best choice for the present study. Additionally, considering the attention to the description of the external characteristics of the literature (e.g., authors, journals, and date of publications), and internal connections (e.g., the co-authorship of the authors and clustering of research topics), a quantitative and comprehensive method, that is, bibliometric analysis was performed. Therefore, the present study aimed to evaluate the depth and breadth of existing research on positive aspects of caregiving in dementia and describe a holistic overview referring to positive aspects of caregiving in the global context, which may contribute to supporting informal caregivers from a positive psychology perspective.

## Methods

The review was developed following the PRISMA Extension for Scoping Reviews [PRISMA-ScR; (31)]. We followed a prior protocol, but it was unpublished and registered. Two steps, scoping review and bibliometric analysis, were performed to cover our study objectives. First, the (32) framework and the updated methodological framework of (30) were adopted for a scoping review. Then, a bibliometric analysis was conducted to analyze the key topic domains, and future study trends using retrieved scoping review results. Quality assessments were not undertaken, as the aim was to examine the full breadth of the literature; consistent with the general aims and methodology of scoping reviews, the critical appraisal is not a required component of the scoping review framework (32).

### Step 1: Scoping review

#### Stage 1: Identifying the research question

The overall research objective of this study was to draw up the current evidence on positive aspects of caregiving in family caregivers with dementia.

The following research questions were identified to answer the objective of this study:

(a) What are the key characteristics of publications, such as date, country, and type of publications on positive aspects of caregiving in dementia?
(b) What kinds of concepts are contained in positive aspects of caregiving and what relevant measurements are applied to assess these conceptions?
(c) What theories are performed to guide the research?
(d) What are the key topic domains covered by the included literature and the potential future trends?

#### Stage 2: Identifying databases and relevant studies

Five electronic databases, including PubMed, CINAHL, PsycINFO, Embase, and Web of Science, were systematically searched from the established date of the library to 11 February 2022. The search strategies combined the subject terms and free words, consisting of terms pertaining to dementia, caregiver, and positive aspects of caregiving. The search strategy of each database was shown in Supplementary material S1. Relevant references of articles and reviews included in this review were manually searched to ensure that all relevant primary studies were contained.

#### Stage 3: Study selection

The review considered the studies in line with the following inclusion and exclusion criteria. Inclusion criteria: (a) studies focused on the positive aspects of caregiving in dementia; (b) caregivers who were informal (unpaid) caregivers of dementia, defined as family members such as spouses, adult–child, and other relatives; (c) settings of studies were community and/or at home, and (d) studies published in English. Exclusion criteria: (a) studies with mixed (paid and unpaid) caregivers of dementia and presented no separate information of family caregivers; (b) studies of individuals with mild cognitive impairment; (c) studies focused on the experience of palliative care, end-of-life or bereavement stage; and (d) conference abstract, letters to the editor, comments, and literature without full text.

All literature searched were imported Endnote X8 to delete duplicates, next, read literature titles, or abstracts for preliminary screening, which included eligible literature and literature that could not be excluded, and then determined the final included literature by reading the full text. Considering that a large amount of literature was involved, the preliminary screening was performed by two researchers simultaneously without back-to-back screening. Before that, two researchers screened the same 50 literature titles, and the consistency of the results was kappa = 0.92. The full-text reading stage was conducted independently by two researchers. When two researchers checked disagreements, they were judged by the rest of the research team.

#### Stage 4: Charting the data

First, a customized data extraction workbook was developed in Microsoft Excel, and a pilot data extraction of 10 pieces of literature was performed by the first author. Later, team members discussed the consistency of data entry and usability of templates and made changes accordingly. Then, formal data extraction was performed by two researchers. Major information extracted contained the following aspects: (a) article characteristics (i.e., author, published date, country, and journal name); (b) study type (i.e., cross-sectional study, longitudinal study, qualitative study, experimental study, mixed methods, review, or others); (c) forms of positive aspects of caregiving and measurements (i.e., meaning, benefit, rewards, self-efficacy, resilience, etc.); (d) theories used in the article; (e) form of dementia (e.g., Alzheimer's disease and all types of dementia); (f) form of family caregiver (i.e., spouse, adult–child, and all types of the family caregiver); and (g) article keywords supplied by the authors or database (when without author-keywords).

#### Stage 5: Collating, summarizing, and reporting the results

A descriptive analysis using frequency and percentage was presented to describe key characteristics of included publications. The research topics were summarized by one researcher with content analysis as the information about the topic was fragmented (33). Phrases and sentences with respect to research objectives and major findings were extracted from the text. Subsequently, the similarities and differences of the analytic units were identified and inductively coded into different themes, and then summarized into the main categories (33). Ultimately, results were constantly reviewed with other team members, and a consensus considering the categorization was reached.

### Step 2: Bibliometric analysis

In addition to the basic descriptive analysis of stage 5 in step 1 scoping review, VOSviewer 1.6.18 was used to draw a network of co-authors, and a network of keywords co-occurrences. Researchers or keywords were represented by nodes in the figure, the size of which indicated the number of published papers, and the lines between nodes indicated co-relationships. The thicker the line, the more connections. For a deeper understanding of the research topic, term frequency analysis of the included literature titles was performed using NVivo11.

## Results

**Figure 1:**
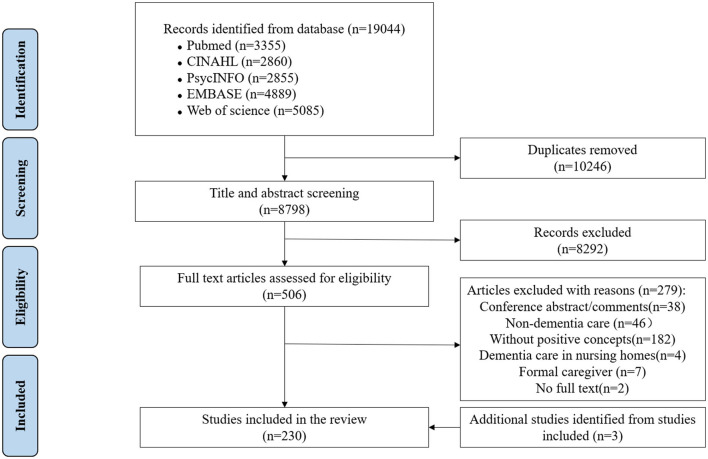
PRISMA flowchart.

A total of 19,044 literature titles were retrieved from five databases; of which 10,246 duplicated literature titles were removed. After a screening of the title and abstract, and an assessment of the full text, 230 full-text articles were included, including 3 additional articles that were searched. Included articles were shown in Supplementary material S2. Figure 1 presents the process of article screening and eligibility.

### Key characteristics and bibliometric properties of the included literature

Table 1 presents the characteristics of publications. Of the 230 publications, most were published in the last decade (*n* = 144, 62.61%). The United States (*n* = 92, 40.00%) contributed most articles, followed by China (*n* = 24, 10.43%), Canada (*n* = 18, 7.83%), and the United Kingdom (*n* = 16, 6.96%). Of the 24 articles in China, 16 (66.7%) were conducted in Hong Kong and Taiwan. Most publications (*n* = 161, 70%) contained all forms of dementia, and a quarter of the articles focused on Alzheimer's disease. Regarding specific caregivers of focus, spouses of dementia (*n* = 19, 8.26%) were the primary concern, with relatively few children, but 86.6% of the literature included all forms of family caregivers. Overall, the majority used quantitative methods (*n* = 162, 70.43%), 11 (4.78%) used a mixed methods design, and 31 (13.48%) employed qualitative methods alone. Most quantitative studies were cross-sectional (*n* = 95, 41.30%), with 26 (11.30%) experimental studies and 13 (5.65%) longitudinal studies. Additionally, 22 (9.57%) publications focused on the development of measurements. Of 32 non-empirical publications, most were reviews, with a further classified as case reports. Twenty-two dissertations were included in the corresponding database, and others were spread across 106 journals. The top five journals were *Aging Ment Health, Gerontologist, Int J Geriatr Psychiatry, Int Psychogeriatr*, and *Dementia (London)*, accounting for 25.8% of the total.

**Table 1:** Summary of key characteristics of included publications.

**Classification**	**Papers (*N* = 230), *n* (%)**
**Country**	
USA	92 (40.00)
China	24 (10.43)
Canada	18 (7.83)
UK	16 (6.96)
Spain	17 (7.39)
Other	63 (27.39)
**Year**	
In the past 10 years (from 2012 to 2022)	144 (62.61)
For the last 20 to 10 years (from 2002 to 2011)	49 (21.30)
Twenty years ago (<2002)	37 (16.09)
**Publication type and study methods**	
Cross-sectional study	95 (41.30)
Quantitative study	31 (13.48)
Experimental study	26 (11.30)
Development of measurements	22 (9.57)
Review	22 (9.57)
Longitudinal study	13 (5.65)
Mixed study	11 (4.78)
Others	10 (4.35)
**Forms of dementia**	
All	161 (70.00)
Alzheimer's disease	65 (28.26)
Others	4 (1.74)
**Forms of caregiver**	
Family caregiver (unclassified)	204 (88.7)
Spouse	19 (8.26)
Adult–child	7 (3.04)
**Journals**	
*Aging Ment Health*	19 (8.26)
*Gerontologist*	14 (6.09)
*Int J Geriatr Psychiatry*	10 (4.35)
*Int Psychogeriatr*	9 (3.91)
*Dementia (London)*	8 (3.48)
Others	170 (73.91)

### Network of researchers and co-authors

**Figure 2:**
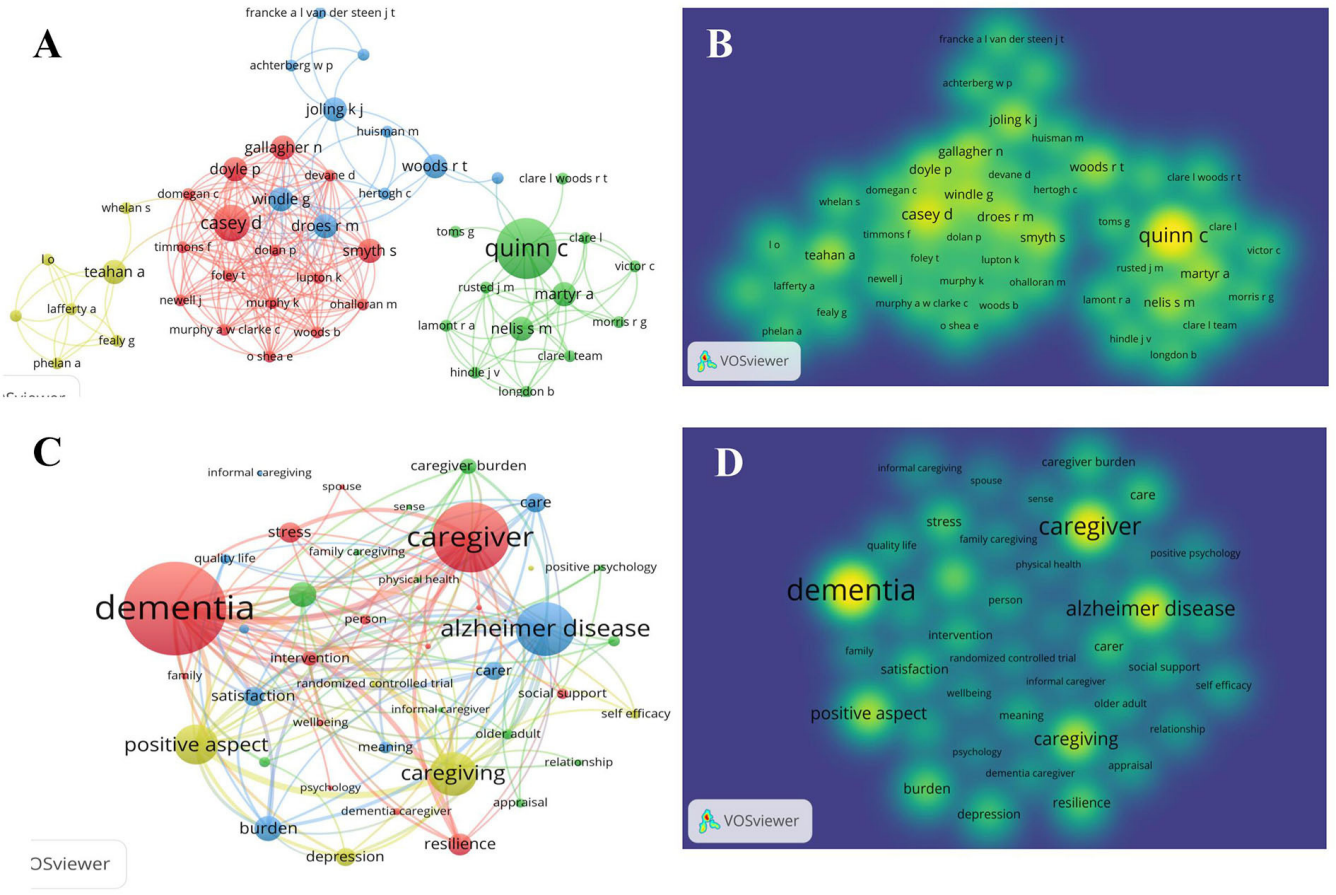
Network analysis of authors and keywords using the density visualization module of VOSviewer software. **(A,B)** Network of authors. **(C,D)** Network of keywords.

Of 230 articles, 710 authors were included, ranging from one author to 19 authors, with an average of 3.08 papers for each author. According to Price's law (34), the core author's publication volume Mp = 0.794 × (NPmax)^1/2^, the author's maximum publication volume (i.e., NPmax) is 19, and the calculation Mp ≈ 3.88, that is, the author who published more than 4 papers was the core author, and there were a total of 8 authors (i.e., Cheng ST, Lam LCW, Zarit SH, Kwok T, Farran CJ, Quinn C, Chan WC, and Peacock S). Figures 2A,B presents the network of co-authors with a large set of connected authors consisting of 46 items. In this network, a total of 4 clusters occurred (cluster 1 = 16 authors, cluster 2 = 13 authors, cluster 3 = 10 authors, and cluster 4 = 7 authors). These four clusters generated co-authorship advantages dominated by Casey D, Quinn C, Joling KJ, and Teahan A, respectively. Additionally, Windle G and Droes RM connected intimated cooperation of cluster 1 and cluster3.

### Network of keywords

Of 230 influential publications, 173 had author-contributed keywords, 26 articles were extracted from the keywords given by the database, and the remaining 32 articles did not find keywords, with a total of 462 unique keywords in this subset of papers. The frequency of these keywords was visually demonstrated in the network of co-occurring keywords. The sociogram reflected terms that occurred 5 times, including 41 keywords. According to Figures 2C,D, the most common keywords (highest in-degree score) were as follows: “dementia (*n* = 101),” “caregiver (*n* = 76),” “Alzheimer's disease (*n* = 58),” “caregiving (*n* = 47),” “positive aspect (*n* = 43),” and “burden (*n* = 27).” Four clusters were classified from the bibliometric mapping generated from the software. Cluster 1 in red included the largest number of items, containing keywords more focused on the resilience and stress of dementia caregivers, and providing support/interventions (dementia, caregiver, resilience, stress, intervention, and social support). Cluster 2 in green contained 12 items focused on the relationship between family caregiver burden and positive aspects (family caregiver, caregiver burden, positive psychology, and relationship). Moreover, the yellow cluster, containing 9 items, grouped terms directly focused on the positive aspects of caregiving (positive aspect, caregiving, depression, self-efficacy, and randomized control trial). Lastly, the blue cluster contained terms more related to positive and negative experiences of caring for Alzheimer's disease (Alzheimer's disease, satisfaction, burden, and meaning). These clusters exhibited an important feature that positive aspects (e.g., positive aspects of caregiving, resilience, and self-efficacy) and negative experiences (e.g., burden and stress) were linked and put together to study.

### Forms of positive aspects of caregiving and measurements

We reviewed quantitative studies or mixed studies that included quantitative elements, collecting a total of 23 types of positive aspects of caregiving and 54 measurements. Positive aspects of caregiving included positive appraisal (*n* = 71), self-efficacy (*n* = 29), satisfaction (*n* = 28), well-being (*n* = 14), gains (*n* = 10), meaning (*n* = 12), resilience (*n* = 10), and competence (*n* = 10). Apart from that, rewards, hope, benefit, gratification, enjoyment, and uplifts were also mentioned in the literature. The measurements most cited were described in Table 2. The Positive Aspects of Caregiving Scale (PACS), developed by (35), was most frequently cited. The original version of the positive aspects of caregiving included 11 items and was subsequently simplified to a nine-item measure, comprising self-affirmation and outlook on life. Additionally, Gain in Alzheimer's Care instrument (GAIN) (37) and the Finding Meaning Through Caregiving Scale (FMTCS) (38) were commonly used by researchers. FMTCS consisted of three dimensions: (a) loss/powerlessness, that is, the negative aspects of caregiving, (b) provisional meaning was conceptualized as finding meaning in a day-to-day caregiving role, and (c) ultimate meaning, which identified caregivers of people with dementia find ultimate meaning in their experience through their spiritual, religious, and philosophical beliefs (38). Loss/powerlessness were reverse-scored for the total scores. GAIN, developed by (37) in an Asian cultural setting through in-depth interviews with caregivers of people with dementia, had 10 items without dimensions. It contained a positive experience for caregivers, and the acquisition of knowledge and skills that were not addressed in PACS. The Revised Scale for Caregiving Self-Efficacy (RSSE) measures three subscales, including obtaining respite, responding to disruptive patient behaviors, and controlling upsetting thoughts (36). Other forms of positive aspects of caregiving and related measurements were shown in Supplementary material S3.

**Table 2:** Description of the measurements most cited included in the study.

**Form of PAC**	**Measurement**	**Description**	**Reliability and validity**
PAC	The Positive Aspects of Caregiving Scale (PACS) (35)	9 items, two subscales, including self-affirmation and outlook on life, initially consisted of 11 items; 5-point Likert scale from 1 (disagree a lot) to 5 (agree a lot); Higher scores indicate greater positive aspects	Internal consistency: Cronbach's α = 0.89; CFA provided a two-factor model fit, with an RMSEA of 0.0592; Positively correlated with well-being, self-reported health, and satisfaction with received social support (r = 0.01 to 0.24, *p* < 0.05); Negatively correlated with burden (r = −0.23, *p* < 0.05)
Self-efficacy	The Revised Scale for Caregiving Self-Efficacy (36)	15 items, 3 subscales, including obtaining respite, responding to disruptive patient behaviors, and controlling upsetting thoughts A Likert scale from 0 to 100 Higher scores reflect greater self-efficacy	Internal consistency: Cronbach's α= 0.75–0.85. Test–retest reliability was α = 0.70–0.76 for the three subscales; CFA provided a three-factor model fit, with a CFI of 0.93; Positively correlated with perceived social support (r = 0.47, *p* < 0.001); Negatively correlated with depression, anxiety, and anger (r = −0.37 to −0.45, *p* < 0.01)
Gains	The Gain in Alzheimer's Care Instrument (GAIN) (37)	10 items without subscale A Likert scale from 0 (disagree a lot) to 4 (agree a lot); Higher scores indicate greater gain	Internal consistency: Cronbach's α = 0.89. Test–retest reliability: α = 0.70 Positively correlated with PACS, DMSS (encouragement and active management) (r = 0.35 to 0.68, *p* < 0.0001); Negatively correlated with ZBI (r = −0.15, *p* = 0.02)
Meaning	The Finding Meaning Through Caregiving Scale (38)	43 items, three subscales, including loss/powerlessness, provisional meaning, and ultimate meaning 5-point Likert scale, 1 (strongly disagree) to 5 (strongly agree) A higher score indicates the greater meaning	Internal consistency: Cronbach's α=0.91. Test–retest reliability α = 0.80 CFA provided strong support for the construct validity of three subscales, with a goodness of fit index of 0.763, and a coefficient of determination of 0.998. Positively correlated with satisfaction, and personal gain (r = 0.39 to 0.58, *p* < 0.01); Negatively correlated with depression, strain, and marital tension (r = −0.35 to −0.64, p < 0.01)

PAC, positive aspects of caregiving; CFA, confirmatory factor analysis; RMSEA, root mean square of approximate error; DMSS, dementia management strategies scale; CFI, comparative fit index.

### Theories used in the included studies

Nearly one-third of the articles (*n* = 64) used explicit theories, models, frameworks, and constructs to guide research. We extracted 20 theories, referring to theoretical frameworks from humanities and social science research, particularly psychology. These theories could be classified into two paradigms, that is, stress coping and positive psychology. Considering the stress and coping theory, 15 articles were referred to Lazarus' stress model, focusing on stress, cognitive appraisal, and coping process (39). The stress process model of caregiving (*n* = 11), proposed by (40), viewed stress as a consequence of an appraisal and coping process facing stressors and resources of caregivers. Moreover, some studies have addressed theories adapted from the stress theory of (39) and/or (40) with background and research purposes. In these studies, positive aspect variables were often considered as an indicator of concern in studies focusing on the negative aspects of caregiving, without a comprehensive framework of positive aspects of caregiving. With the concern of positive psychology, relevant theories were also used to guide positive aspects of caregiving in dementia, which offered an alternative to the stress theory. Resilience theory and self-efficacy theory were mostly performed to guide studies considering specific elements of positive aspects of caregiving (i.e., resilience and self-efficacy) (*n* = 7). The positive psychology framework, proposed by (41) was used in four studies to achieve a greater understanding of positive emotion and experience of dementia caregiving. Theories were shown in Supplementary material S4.

### Key topics of included studies

**Figure 3:**
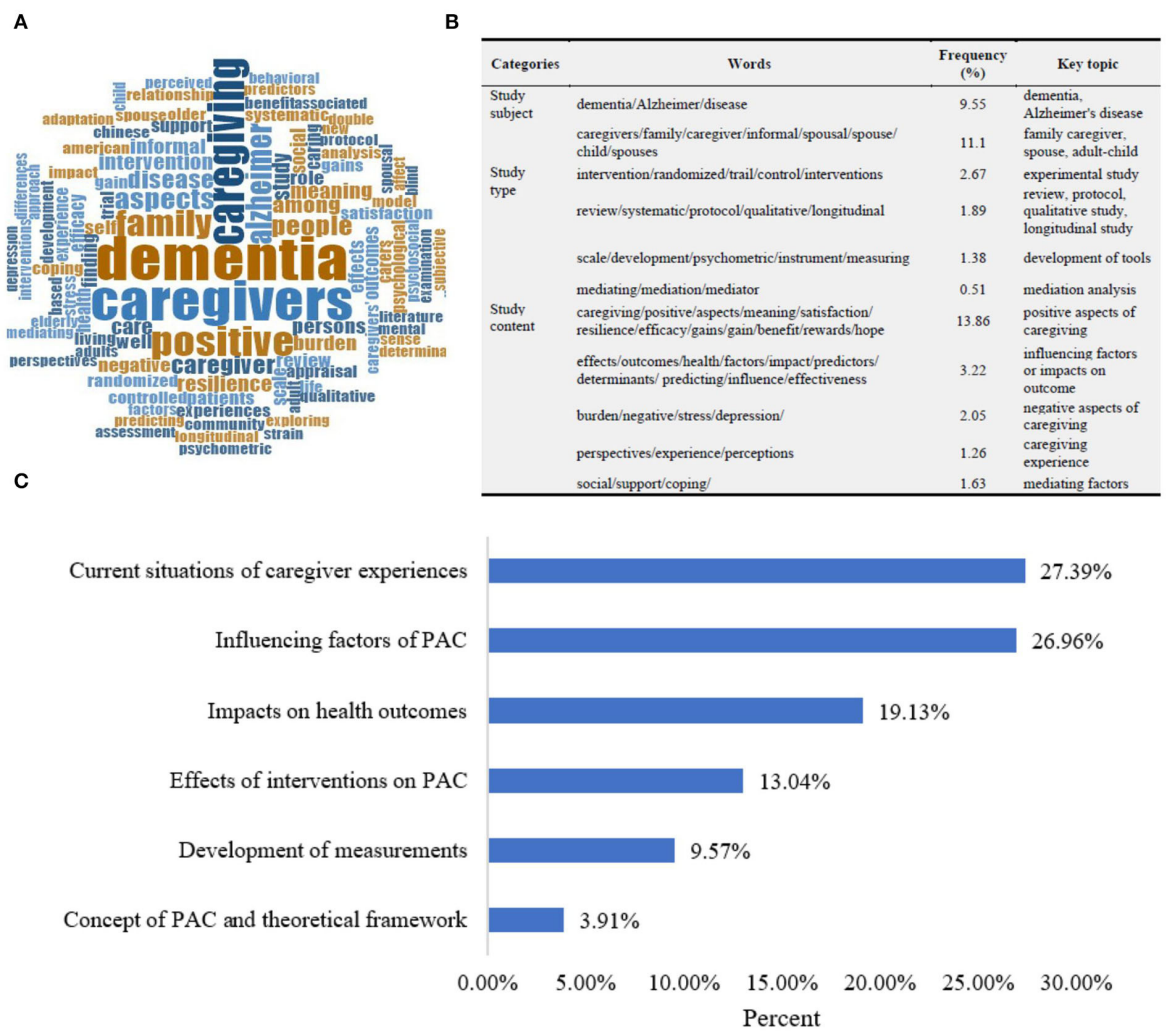
Word cloud analysis of titles **(A,B)** and research key topics of included literature **(C)**.

We identified key topics through word cloud analysis of the title and content analysis of included study objectives. Considering world cloud analysis, we removed caregiver, caregiving, carer, and other words that did not distinguish the research topic and included 607 words to generate a word cloud map. The higher the frequency, the larger the word (Figure 3A). According to the content of the documents retrieved in each database for each high-frequency word, combined with professional knowledge, it was inferred from the analysis of the study type, study subjects, and study content (Figure 3B). Some studies have focused on spouses or adult–child with dementia; most studies included various types of family caregivers. The subjects of care mainly included various types of dementia and specific type (i.e., Alzheimer's disease). These characteristics were in accordance with the findings in Table 1. Regarding the study content, in agreement with the network of keywords, the study explored the influencing factors and impacts on health outcomes. Apart from positive aspects of caregiving, studies also combined the negative aspects of caregiving and identified their links.

Our inductive content analysis of the research objectives identified several key topic and subtopic areas (Figure 3C): (1) current situations of caregiver experiences, focusing on the coexistence of positive and negative aspects of caregiving, how and why to emerge; (2) influencing factors, which could be summed up as stressors (i.e., dementia-related characteristics, caregiver-related characteristics, and context factors) and coping resources (i.e., individual internal resources, including self-efficacy, resilience, and coping strategies; external resources, that is, social support and services available); (3) positive aspects of caregiving as predictors or mediators of health outcomes (e.g., well-being and quality of life), mainly using path analysis based on cross-sectional data; (4) development of measurements (validation study of relevant tools, etc.); (5) interventions on positive aspects of caregiving, comprising psychosocial intervention, psychoeducational intervention, social support, etc.; and (6) concept and theoretical framework identified by narrative, integrative, critical review or Delphi study.

## Discussion

This review demonstrates the nature and range of scientific literature on positive aspects of caregiving in dementia. To our knowledge, this is the first study to comprehensively describe characteristics, key topic domains, and gaps identified at the international level using scoping review nested bibliometric analysis. The discussion of the results involves four aspects: (a) nature and extent of literature. (b) concepts of positive aspects of caregiving. (c) theories used in the studies, and (d) research topic domain.

### Nature and extent of literature

We performed an extensive search of the literature in the review, and a large body of literature was available. Overall, literature on positive aspects of caregiving in dementia has been on the rise since positive psychology emerged in 2000 (42), especially in the last decade. Massive previous studies mainly have focused on the negative aspects of caregiving (6, 7, 43). With the development of positive psychology, the positive feeling of caregiving has become a new hot topic, which gives a more comprehensive understanding of the caregiver experience. High-income countries/regions, such as the United States, Canada, the United Kingdom, Spain, and Hong Kong in China, predominantly made contributions to publications, reflecting general trends previously identified in positive aspects of caregiving (25). Moreover, it suggests the paucity of studies in medium-income countries/regions and even low-income countries, but caregivers in these countries or regions may face greater challenges and threats because of inadequate service support. Positive aspects of caregiving referred to a wide range of research types, covering quantitative, qualitative, cross-sectional, experimental studies, and so on. Qualitative research is favored, which is also in line with the method that can dig into the experience of caregivers. A quarter of the articles focused on Alzheimer's disease, probably because over two-thirds of dementia is Alzheimer's disease, which means that it is convenient to recruit research subjects (44). Moreover, findings display that nearly 80% of individuals with Alzheimer's disease suffered more severe and frequent neuropsychiatric symptoms compared to other forms of dementia (45). In other words, caregivers with Alzheimer's disease experience stronger stressors, a feature that must attract research attention. Lastly, this review can be considered as a surrogate to identify targeted journals and seek international co-authors in the field.

### Concepts and relating measurements

Since a concept like positive aspects of caregiving is essentially multidimensional, currently, it is not clear what specific constructs comprise positive aspects of caregiving, without a unified definition of its connotation and extension. Therefore, this review included many other positive psychological terms as keywords for database search. In the literature, terms like self-efficacy, satisfaction, well-being, gains, meaning, resilience, and competence are certain aspects of positive care outcomes; however, they cannot perfectly reflect the general meaning of positive aspects of caregiving. These positive psychological terms may be the influencing factors of positive aspects of caregiving (e.g., resilience, self-efficacy, etc.) or the positive impacts of positive aspects of caregiving (e.g., well-being, satisfaction, etc.) (25). The varied conceptual and operational terms lead to difficulty to compare and integrate outcomes across studies. Thus, it is needed to develop an internationally accepted definition of positive aspects of caregiving (20) identified four key domains of positive aspects of caregiving from the personal to dyadic and family levels, which would provide a guide to future directions, but a clear research definition of positive aspects of caregiving seems to require further conceptualization. With an in-depth understanding of the concept of positive aspects of caregiving, more and more scholars recognized the limitations of current assessment tools. PACS is the most widely used due to its simple item, psychometric advantage, and availability in various languages. Nevertheless, no item is associated with mutuality in a dyadic relationship and family cohesion. Caregiving is an interactive process between the caregiver and dementia, and a process that involves the families. Focusing on the positive aspects of caregiving at the dyadic and family is indeed essential to understand mechanisms underlying the health-beneficial effects of dyadic and family effects processes (46). Moreover, self-growth did not reflect gains in caregiving skills. Other measurements were also selected according to the authors' definition of positive aspects of caregiving in the literature. Currently, there is no comprehensive-existing measurement to reflect positive aspects of caregiving due to the lack of unified connotation of positive aspects of caregiving.

### Theories used in the studies

According to theories retrieved from articles, two orientations were found in the development of research frameworks. The stress and coping model is the most classic framework (40). Caregiving with dementia is the process of coping with various stressors, such as care dependency, neuropsychiatric symptoms, etc. While facing stressors, individuals experience primary appraisal and secondary appraisal of whether coping resources matched demands (39). The concept of positive aspects of caregiving is regarded as a successful outcome in coping with difficulties and challenges. Despite this, the study using the stress and coping model weakens the generation process of positive aspects, and the negative responses are widely verified. The focus on the positive aspects of caregiving in recent years may spark a new research trend, which is where the value of this review lies. The present findings showed that another orientation of positive psychology mentioned most frequently is resilience theory. Resilience is a construct connecting an individual's maintenance of positive adaptation when facing significant adversity, reflecting the interaction between risk factors and protective factors (47). Nevertheless, articles guided by the resilience theory focused on a specific concept of positive aspects of caregiving, “resilience,” and so did other theories on positive variables (e.g., self-efficacy theory) (48) proposed a protocol for developing a theoretical model, based on the stress and coping model and meaning-making paradigm, to explore how influencing factors affect positive aspects of caregiving. It helps further understand the process of how caregivers obtain positive experiences out of the overwhelming caregiving stress. Since negative aspects, such as perceived stress and caregiver burden, coexist with positive aspects of caregiving (12), meanwhile, previous studies have identified that caregiver burden was negatively related to positive aspects of caregiving (49, 50). It is necessary to explore how these negative variables affect positive aspects of caregiving. In the future, the theoretical framework focusing on the positive aspects of caregiving deserves further study.

### Research topic domain

Not surprisingly, most of the studies were directly associated with the current situation of caregiving experience, influencing factors, and complications. Discovering the essence and meanings of the caregiving experience plays the first-step role in the research topic, which is the reason why numerous studies have documented the coexistence of positive and negative experiences of caregiving (51) conducted a systematic review to describe the holistic experience including the positive and negative aspects underlying the caregiver with dementia role. Subsequently, exploring the influencing factors of positive aspects of caregiving is the basis, thereby contributing to the development of interventions. In line with the discussion on the aspect of theories most cited, influencing factors were classified as stressors and coping resources based on the stress coping theory. Thus, reducing stressors and enhancing coping resources are important directions for the development of interventions. Currently, the research on the influencing factors and impacts of positive aspects of caregiving is blooming all over the world. It has been studied from various perspectives and begun to gradually analyze the relationship and influencing pathway between factors and/or outcomes. Nevertheless, compared with predictors of negative aspects, far fewer predictors of positive aspects are being identified. A longitudinal study examined antecedents and outcomes of positive aspects (i.e., enrichment) using the structural equation model (18). Other path analysis studies are mainly based on cross-sectional data, which cannot determine the causal relationship (52, 53). A narrative synthesis identified factors correlated to a sense of competence from both positive and negative insights (24, 25) evidenced that the concept of positive aspects of caregiving was negatively associated with depressive symptoms and burden, inversely, positively relating variables reflecting well-being (i.e., mental health, quality of life, and satisfaction with life). Exploring the effectiveness of interventions on positive aspects of caregiving is of significance, and this topic is progressively expanded. Psychosocial and psychoeducational interventions are commonly applied. Psychosocial intervention is a multicomponent intervention that is concerned with general information and education support, caregiving skills training, and problem-solving, and cognitive therapy, stress relaxation, psychotherapy, and counseling (54). Large national projects, such as the Resources for Enhancing Alzheimer's Caregiver Health (REACH) research and Research to Assess Policies and Strategies for Dementia in the Young (RHAPSODY) (55–57), were distributed underway in England, France, etc. Additionally, promoting programs of the targeted form of positive aspects of caregiving, such as benefits, resilience, and self-efficacy, was also developing (58–60). Studies focused on the structured education of the transmission of knowledge and skills regarding dementia and caregiving-related issues were included in the psychoeducational intervention (61). For instance, a 10-week psychoeducational program enhanced the positive aspects of care (62). While performing narrative synthesis, we found that the term “psychosocial” and “psychoeducational” were not easy to identify. This review provided a general perspective of interventions, which needs to be clearly defined in future systematic reviews and meta-analyses to evaluate their effectiveness. These researches are complex interventions, including a variety of interactive components (63). They mainly focus on the effectiveness and pay little attention to how and why interventions occur or not, yet this is the way forward for complex interventions (64).

### Strengths and limitations

Although positive aspects of caregiving in dementia have been extensively explored in the last decade, a paucity of comprehensive reviews was conducted to examine the extent, nature, and gaps in the existing literature, especially based on a holistic view. This review, based on Arksey and O'Malley's framework, is the first to comprehensively map the literature on the positive aspects of caregiving. Apart from key characteristics of literature (e.g., country, year, journals, etc.), we performed bibliometric analysis to examine key topics and identified gaps in research to guide future directions on positive aspects of caregiving in dementia. Meanwhile, inductive thematic analysis of research objectives and word cloud analysis of title were also conducted. Additionally, the extensive literature inclusion, including quantitative articles, qualitative articles, reviews, and protocols, was also among the strengths of this study. Compared with other single-database bibliometric analyses, the systematic search and comprehensive literature screening of five databases avoided some missed records.

Several limitations on methodology should be addressed when understanding the results of this review. First, while we developed and followed a protocol for the review, this protocol was unpublished. Second, despite our comprehensive database search, some literature may have been missed, only English literature included may exacerbate this limitation. Then, network analysis of keywords was conducted only in articles with author-provided keywords, and 32 articles without author-provided keywords were not been included, which may affect the clusters of research topics. To support this, inductive thematic analysis of research objectives and word cloud analysis of title were performed. It is of benefit to contain proceeding abstracts in the updated search, given most novel topics firstly emerged in the academic conference exchange. This review excluded the conference abstracts because of the limited information that could be extracted. This review did not summarize the influencing factors of positive aspects of caregiving and the elements contained in positive aspects of caregiving interventions, which can be conducted as specific systematic reviews in the future.

## Conclusion

Given the growth in the aging population worldwide, an increasing incidence of dementia and family caregivers' challenges may be expected. This may impact stress-related health problems and quality of life. Although there is currently a large body of evidence focusing on dementia caregiver interventions (61, 65, 66), this review provides us a glimpse of future trends in developing interventions from positive aspects of caregiving, as indicated by the findings of this study. Although the concept of positive aspects of caregiving is not explicitly understood, an international consensus on the definition of positive aspects of caregiving, development of a comprehensive measurement, and construction of a comprehensive theoretical framework regarding antecedents and consequences of positive aspects of caregiving are recommended to guide caregiver practice and provide caregiver support. Future studies should aim to further develop effective interventions and identify how and why interventions work or not.

## Data availability statement

The data presented in the study is included in the article/Supplementary material, further inquiries can be directed to the corresponding author/s.

## Author contributions

JW: writing—original draft. WL and BY: writing—review. QZ, YL, and MX: writing—review and editing. JW, XL, WL, and BY: screening literature. JW, XL, WL, BY, and MX: conceptualization, methodology, and software. All authors contributed to the article and approved the submitted version.

## Funding

This study was funded by the National Key Research and Development Program of China (2020YFC2005900), Guangzhou Concord Medical Humanities Research and Education Fund (23000-3050070), and CQMU Program for Youth Innovation in Future Medicine (W129).

## Conflict of interest

The authors declare that the research was conducted in the absence of any commercial or financial relationships that could be construed as a potential conflict of interest.

## Publisher's note

All claims expressed in this article are solely those of the authors and do not necessarily represent those of their affiliated organizations, or those of the publisher, the editors and the reviewers. Any product that may be evaluated in this article, or claim that may be made by its manufacturer, is not guaranteed or endorsed by the publisher.

## References

[1] WHO. Dementia. (2021). Available online at: https://www.who.int/news-room/fact-sheets/detail/dementia (accessed September 02, 2021).

[2] Lee K Puga F Pickering CEZ Masoud SS White CL. Transitioning into the caregiver role following a diagnosis of Alzheimer's disease or related dementia: a scoping review. Int J Nurs Stud. (2019) 96:119–31. 10.1016/j.ijnurstu.2019.02.007 30851954

[3] Wang H Li T Barbarino P Gauthier S Brodaty H Molinuevo JL. Dementia care during COVID-19. Lancet. (2020) 395:1190–1. 10.1016/S0140-6736(20)30755-8 32240625 PMC7146671

[4] Aranda MP Kremer IN Hinton L Zissimopoulos J Whitmer RA Hummel CH. Impact of dementia: health disparities, population trends, care interventions, and economic costs. J Am Geriatr Soc. (2021) 69:1774–83. 10.1111/jgs.17345 34245588 PMC8608182

[5] Alzheimer's Disease International. World Alzheimer Report 2018: Global Estimates of Informal Care. (2018). Available online at: https://www.alzint.org/u/global-estimates-of-informal-care.pdf (accessed June 04, 2018).

[6] Oh ES Rabins PV. Dementia. Ann Intern Med. (2019) 171:Itc33–48. 10.7326/aitc201909030 31476229

[7] Wu-Chung EL Leal SL Denny BT Cheng SL Fagundes CP. Spousal caregiving, widowhood, and cognition: a systematic review and a biopsychosocial framework for understanding the relationship between interpersonal losses and dementia risk in older adulthood. Neurosci Biobehav Rev. (2022) 134:104487. 10.7326/AITC201909030 34971701 PMC8925984

[8] Gentry MT Lapid MI Syrjanen J Calvert K Hughes S Brushaber D. Quality of life and caregiver burden in familial frontotemporal lobar degeneration: analyses of symptomatic and asymptomatic individuals within the LEFFTDS cohort. Alzheimers Dement. (2020) 16:1115–24. 10.1002/alz.12095 32656921 PMC7534513

[9] Chekani F Pike J Jones E Husbands J Khandker RK. Impact of dementia-related behavioral symptoms on healthcare resource use and caregiver burden: real-world data from Europe and the United States. J Alzheimers Dis. (2021) 81:1567–78. 10.3233/JAD-201483 34057080 PMC8293640

[10] Tay R Tan JYS Hum AYM. Factors associated with family caregiver burden of home-dwelling patients with advanced dementia. J Am Med Dir Assoc. (2021) 23:1248–56. 10.1016/j.jamda.2021.09.012 34634231

[11] Dickinson C Dow J Gibson G Hayes L Robalino S Robinson L. Psychosocial intervention for carers of people with dementia: What components are most effective and when? a systematic review of systematic reviews. Int Psychogeriatrics. (2016) 29:31–3. 10.1017/S1041610216001447 27666669

[12] Ar Y Karanci AN. Turkish adult children as caregivers of parents with Alzheimer's disease: perceptions and caregiving experiences. Dementia. (2019) 18:882–902. 10.1177/1471301217693400 28201932

[13] Williams IC. Emotional health of Black and White dementia caregivers: a contextual examination. J Gerontol B Psychol Sci Soc Sci. (2005) 60:287–P295. 10.1093/geronb/60.6.P287 16260702

[14] Lloyd J Patterson T Muers J. The positive aspects of caregiving in dementia: a critical review of the qualitative literature. Dementia. (2016) 15:1534–61. 10.1177/1471301214564792 25547208

[15] Autio T Rissanen S. Positive emotions in caring for a spouse: a literature review. Scand J Caring Sci. (2018) 32:45–55. 10.1111/scs.12452 28543793

[16] Richards A. Finding meaning in caregiving, well-being, and spousal caregivers of people with dementia (Ph.D. thesis). University of Surrey, Guildford, United Kingdom. (2011).

[17] Johansson MF McKee KJ Dahlberg L Summer Meranius M Williams CL Marmstål Hammar L. Negative impact and positive value of caregiving in spouse carers of persons with dementia in Sweden. Int J Environ Res Public Health. (2022) 19:31788. 10.3390/ijerph19031788 35162811 PMC8835239

[18] Morimoto H. Takebayashi Y. Antecedents and outcomes of enrichment among working family caregivers of people with dementia: a longitudinal analysis. J Gerontol Series B-Psychol Sci Soc Sci. (2021) 76:1060–70. 10.1093/geronb/gbaa183 33099602 PMC8200353

[19] Kobiske KR Bekhet AK. Resilience in caregivers of partners with young onset dementia: a concept analysis. Issues Mental Health Nurs. (2018) 39:411–19.: 10.1080/01612840.2017.1400625 29370559 10.1080/01612840.2017.1400625

[20] Yu DSF Cheng ST Wang JF. Unravelling positive aspects of caregiving in dementia: An integrative review of research literature. Int J Nurs Stud. (2018) 79:1–26. 10.1016/j.ijnurstu.2017.10.008 29128685

[21] Pione RD Spector A Cartwright AV Stoner C. R. A psychometric appraisal of positive psychology outcome measures in use with carers of people living with dementia: a systematic review. Int Psychogeriatrics. (2021) 33:385–404. 10.1017/S1041610220003464 33081861

[22] Petriwskyj A Parker D O'Dwyer S Moyle W Nucifora N. Interventions to build resilience in family caregivers of people living with dementia: a comprehensive systematic review. JBI Database System Rev Implement Rep. (2016) 14:238–73. 10.11124/JBISRIR-2016-002555 27532659

[23] Teahan A Lafferty A McAuliffe E Phelan A O'Sullivan L O'Shea D. Resilience in family caregiving for people with dementia: a systematic review. Int J Geriatr Psychiatry. (2018) 33:1582–95. 10.1002/gps.4972 30230018

[24] Stansfeld J Crellin N Orrell M Wenborn J Charlesworth G Vernooij-Dassen M. Factors related to sense of competence in family caregivers of people living with dementia in the community: a narrative synthesis. Int Psychogeriatrics. (2019) 31:799–813. 10.1017/S1041610218001394 30466499 PMC6398586

[25] Quinn C Toms G. Influence of positive aspects of dementia caregiving on caregivers' well-being: a systematic review. Gerontologist. (2019) 59:E584–96. 10.1093/geront/gny168 30597058

[26] Han A. Interventions for attitudes and empathy toward people with dementia and positive aspects of caregiving: a systematic review and meta-analysis. Res Aging. (2020) 42:72–82. 10.1177/0164027519884766 31713456

[27] Pleasant M Molinari V Dobbs D Meng H Hyer K. Effectiveness of online dementia caregivers training programs: a systematic review Geriatric Nursing. (2020) 41:921–35. 10.1016/j.gerinurse.2020.07.004 32703628

[28] Daudt HM van Mossel C Scott SJ. Enhancing the scoping study methodology: a large, inter-professional team's experience with Arksey and O'Malley's framework. BMC Med Res Methodol. (2013) 13:48. 10.1186/1471-2288-13-48 23522333 PMC3614526

[29] Peters MD Godfrey CM Khalil H McInerney P Parker D Soares CB. Guidance for conducting systematic scoping reviews. Int J Evid Based Healthc. (2015) 13:141–46. 10.1097/XEB.0000000000000050 26134548

[30] Levac D Colquhoun H O'Brien KK. Scoping studies: advancing the methodology. Implement Sci. (2010) 5:69. 10.1186/1748-5908-5-69 20854677 PMC2954944

[31] Tricco AC Lillie E Zarin W O'Brien KK Colquhoun H Levac D. PRISMA Extension for Scoping Reviews (PRISMA-ScR): checklist and explanation. Ann Intern Med. (2018) 169:467–73. 10.7326/M18-0850 30178033

[32] Arksey H O'Malley L. Scoping studies: towards a methodological framework. Int J Soc Res Methodol. (2005) 8:19–32. 10.1080/1364557032000119616

[33] Elo S Kyngäs H. The qualitative content analysis process. J Adv Nurs. (2008) 62:107–15. 04569.x. 10.1111/j.1365-2648.2007.04569.x 18352969

[34] Hu Z Zhang Y. Analysis of core authors and extended core authors based on price law and comprehensive index method. J Southwest Univer Nationalities (Natural Science Edition). (2016) 42:351–4.

[35] Tarlow BJ Wisniewski SR Belle SH Rubert M Ory MG Gallagher-Thompson D. Positive aspects of caregiving: contributions of the REACH Project to the development of new measures for Alzheimer's caregiving. Res Aging. (2004) 26:429–53. 10.1177/0164027504264493

[36] Steffen AM McKibbin C Zeiss AM Gallagher-Thompson D Bandura A. The revised scale for caregiving self-efficacy: reliability and validity studies. J Gerontol B Psychol Sci Soc Sci. (2002) 57:74–86. 10.1093/geronb/57.1.P74 11773226

[37] Yap P Luo N Ng WY Chionh HL Lim J Goh J. Gain in Alzheimer care INstrument–a new scale to measure caregiving gains in dementia. Am J Geriatr Psychiatry. (2010) 18:68–76. 10.1097/JGP.0b013e3181bd1dcd 20094020

[38] Farran CJ Miller BH Kaufman JE Donner E Fogg L. Finding meaning through caregiving: Development of an instrument for family caregivers of persons with Alzheimer's disease. J Clin Psychol. (1999) 55:1107–25. 10.1002/(sici)1097-4679(199909)55:9<1107::aid-jclp8>3.0.co;2-v 10576325

[39] Lazarus RS Folkman S. Stress, Appraisal, and Coping. New York, NY: Springer (1986).

[40] Pearlin LI Mullan JT Semple SJ Skaff MM. Caregiving and the stress process: an overview of concepts and their measures. Gerontologist. (1990) 30:583–94. 10.1093/geront/30.5.583 2276631

[41] Seligman ME Steen TA Park N Peterson C. Positive psychology progress: empirical validation of interventions. Am Psychol. (2005) 60:410–21. 10.1037/0003-066X.60.5.410 16045394

[42] Seligman MEP Csikszentmihalyi M. Positive psychology. Ame Psychologist. (2000) 55:5–14. 10.1037//0003-066x.55.1.5 11392865

[43] Karnatz T Monsees J Wucherer D Michalowsky B Zwingmann I Halek M. Burden of caregivers of patients with frontotemporal lobar degeneration - a scoping review. Int Psychogeriatr. (2021) 33:891–911. 10.1017/S1041610219000176 30982478

[44] Chan KY Wang W Wu JJ Liu L Theodoratou E Car J. Epidemiology of Alzheimer's disease and other forms of dementia in China, 1990-2010: a systematic review and analysis. Lancet. (2013) 381:2016–23. 10.1016/S0140-6736(13)60221-4 23746902

[45] Lyketsos CG Lopez O Jones B Fitzpatrick AL Breitner J DeKosky S. Prevalence of neuropsychiatric symptoms in dementia and mild cognitive impairment: results from the cardiovascular health study. JAMA. (2002) 288:1475–83. 10.1001/jama.288.12.1475 12243634

[46] Wuttke-Linnemann A Baake R Fellgiebel A. Dyadic wind of change: new approaches to improve biopsychological stress regulation in patients with dementia and their spousal caregivers. J Alzheimers Dis. (2019) 68:1325–37. 10.3233/JAD-181025 30909228

[47] Luthar SS Cicchetti D Becker B. The construct of resilience: a critical evaluation and guidelines for future work. Child Dev. (2000) 71:543–62. 10.1111/1467-8624.00164 10953923 PMC1885202

[48] Yu DSF Cheng ST Kwok T. Developing and testing of an integrative theoretical model to predict positive aspects of caregiving among family caregivers of persons with dementia: A study protocol. J Adv Nurs. (2021) 77:401–10. 10.1111/jan.14561 33068058

[49] Abdollahpour I Nedjat S Salimi Y. Positive aspects of caregiving and caregiver burden: a study of caregivers of patients with dementia. J Geriatr Psychiatry Neurol. (2018) 31:34–8. 10.1177/0891988717743590 29187025

[50] Wang ZX Ma CY Han HJ He RL Zhou LY Liang RF. Caregiver burden in Alzheimer's disease: Moderation effects of social support and mediation effects of positive aspects of caregiving. Int J Geriatr Psychiatry. (2018) 33:1198–206. 10.1002/gps.4910 29856091

[51] Lindeza P Rodrigues M Costa J Guerreiro M Rosa MM. Impact of dementia on informal care: a systematic review of family caregivers' perceptions. BMJ Support Palliat Care. (2020) 1–12. 10.1136/bmjspcare-2020-002242 33055092

[52] Yu H Wu L Chen S Wu Q Yang Y Edwards H. Caregiving burden and gain among adult-child caregivers caring for parents with dementia in China: the partial mediating role of reciprocal filial piety. Int Psychogeriatr. (2016) 28:1845–55. 10.1017/S1041610216000685 27255608

[53] Wawrziczny E Antoine P Doba K. Modeling the distress of adult-child caregivers of people with dementia: the mediating role of self-efficacy. J Alzheimers Dis. (2021) 84:855–67. 10.3233/JAD-210624 34602477

[54] Cooke DD McNally L Mulligan KT Harrison MJ Newman SP. Psychosocial interventions for caregivers of people with dementia: a systematic review. Aging Ment Health. (2001) 5:120–35. 10.1080/13607863.2001.11070746 11511059

[55] Patterson TR. African Americans and the Alzheimer's Caregiving experience: Differential intervention efficacy within the stress process model of caregiving (Ph.D. thesis). North Carolina State University, Raleigh, NC, United States. (2015).

[56] Metcalfe A Jones B Mayer J Gage H Oyebode J Boucault S. Online information and support for carers of people with young-onset dementia: A multi-site randomised controlled pilot study. Int J Geriatr Psychiatry. (2019) 34:1455–64. 10.1002/gps.5154 31111516

[57] Asiret GD Yilmaz CK Kasar KS. Investigation of the effects of interventions made according to the progressively lowered stress threshold model on the care outcomes of Alzheimer patients and their families: a randomized clinical trial. Psychogeriatrics. (2021) 21:738–48. 10.1111/psyg.12734 34233376

[58] Cheng ST Mak EPM Kwok T Fung H Lam LCW. Benefit-Finding intervention delivered individually to alzheimer family caregivers: longer-term outcomes of a randomized double-blind controlled trial. J Gerontol B Psychol Sci Soc Sci. (2020) 75:1884–93. 10.1093/geronb/gbz118 31556447

[59] Doyle P Gallagher N Smyth S Casey D. Exploring the feasibility and acceptability of a Comprehensive Resilience-building psychosocial intervention (CREST) for people with dementia in the community: A nonrandomised feasibility study. Int Psychogeriatrics. (2020) 32:101. 10.1017/S1041610220002410 PMC7667740 33292667

[60] Cheng ST Chan WC Fung HH Lam LCW. Self-efficacy in controlling upsetting thoughts, but not positive gains, mediates the effects of benefit-finding group intervention for Alzheimer family caregivers. Psychol Aging. (2021) 1–10. 10.1037/pag0000654 34843329

[61] Walter E Pinquart M. How effective are dementia caregiver interventions? an updated comprehensive meta-analysis. Gerontologist. (2020) 60:609–19. 10.1093/geront/gnz118 33226434

[62] Paul C Teixeira L Duarte N Pires CL Ribeiro O. Effects of a community intervention program for dementia on mental health: the importance of secondary caregivers in promoting positive aspects and reducing strain. Community Ment Health J. (2019) 55:296–303. 10.1007/s10597-018-0345-6 30361913

[63] Corry M Clarke M While AE Lalor J. Developing complex interventions for nursing: a critical review of key guidelines. J Clin Nurs. (2013) 22:2366–86. 10.1111/jocn.12173 23551526

[64] Skivington K Matthews L Simpson SA Craig P Baird J Blazeby JM. A new framework for developing and evaluating complex interventions: update of medical research council guidance. BMJ. (2021) 374:n2061. 10.1136/bmj.n2061 34593508 PMC8482308

[65] Leng M Zhao Y Xiao H Li C Wang Z. Internet-Based supportive interventions for family caregivers of people with dementia: systematic review and meta-analysis. J Med Internet Res. (2020) 22:e19468. 10.2196/19468 32902388 PMC7511858

[66] Sun Y Ji M Leng M Li X Zhang X Wang Z. Comparative efficacy of 11 non-pharmacological interventions on depression, anxiety, quality of life, and caregiver burden for informal caregivers of people with dementia: a systematic review and network meta-analysis. Int J Nurs Stud. (2022) 129:104204. 10.1016/j.ijnurstu.2022.104204 35247788

