# Supplementary material for: The positive aspects of caregiving in dementia: A scoping review and bibliometric analysis
Source: Front Public Health. 2022 Sep 14;10:985391. doi: 10.3389/fpubh.2022.985391 (PMC9515975; doi:10.3389/fpubh.2022.985391)
Supplement: Supplementary file 1 [file Data_Sheet_1.docx]

Supplementary Material

**Supplementary S1: Search strategies**

**Supplementary S2: Included articles in this review (n=230)**

**Supplementary S3: Forms of positive aspects of caregiving and related measurements**

**Supplementary S4: Theories most cited in the studies**

**Supplementary S1: Search strategies**

| 1. Pubmed | | |
| --- | --- | --- |
| #1 | Search "Dementia"[MeSH Terms] | |
| #2 | Search (dement*[Title/Abstract]) OR alzheimer*[Title/Abstract] | |
| #3 | #1 OR #2 | |
| #4 | Search Caregivers [MeSH Terms] | |
| #5 | Search (caregiv*[Title/Abstract]) OR (carer*[Title/Abstract]) | |
| #6 | #4 OR #5 | |
| #7 | Search Psychology, Positive [MeSH Terms] | |
| #8 | (((((((((((((((positive aspect[Title/Abstract]) OR (positive experience[Title/Abstract])) OR (positive perceptions[Title/Abstract])) OR (positive appraisal[Title/Abstract])) OR (Positive impact[Title/Abstract])) OR (reward[Title/Abstract])) OR (gain*[Title/Abstract])) OR (satisfaction*[Title/Abstract])) OR (benefit*[Title/Abstract])) OR (uplift*[Title/Abstract])) OR (meaning*[Title/Abstract])) OR (enjoyment*[Title/Abstract])) OR (pleasure[Title/Abstract])) OR (growth[Title/Abstract])) OR (hope[Title/Abstract])) OR (gratification[Title/Abstract]) | |
| #9 | #7 OR #8 | |
| #10 | #3 AND #6 AND #9 | |
| #11 | (English[Language]) AND #3 AND #6 AND #9  3355 results for search #11 | |
| 2.CINAHL | | |
| S1 | (MH "Dementia+") | |
| S2 | TI (dementia* OR alzheimer*) OR AB dementia* OR AB alzheimer* | |
| S3 | S1 OR S2 | |
| S4 | (MH "caregivers") |  |
| S5 | TI caregiv* OR TI carer* OR AB caregiv* OR AB carer* |  |
| S6 | S4 OR S5 |  |
| S7 | (MH "psychologie positive") |  |
| S8 | TI positive aspect OR positive experience OR positive perceptions OR positive appraisal OR positive impact OR reward OR gain* OR satisfaction* OR benefit* OR uplift* OR meaning* OR enjoyment* OR pleasure OR growth OR hope OR gratification |  |
| S9 | AB positive aspect OR positive experience OR positive perceptions OR positive appraisal OR positive impact OR reward OR gain* OR satisfaction* OR benefit* OR uplift* OR meaning* OR enjoyment* OR pleasure OR growth OR hope OR gratification |  |
| S10 | S7 OR S8 OR S9 |  |
| S11 | S3 AND S6 AND S10, limiters-English language  2860 results for search #13 |  |
| 3. PsychINFO | |  |
| S1 | (MA "Dementia") |  |
| S2 | TI Dementia* OR TI alzheimer* OR AB dementia* OR AB alzheimer* |  |
| S3 | S1 OR S2 |  |
| S4 | (MA "caregivers") |  |
| S5 | TI caregiv* OR TI carer* OR AB caregiv* OR AB carer* |  |
| S6 | S4 OR S5 |  |
| S7 | (MA"psychologie positive) |  |
| S8 | TI psychologie positive OR TI (positive aspect OR positive experience OR positive perceptions OR positive appraisal OR positive impact OR reward OR gain* OR satisfaction* OR benefit* OR uplift* OR meaning* OR enjoyment* OR pleasure OR growth OR hope OR gratification) |  |
| S9 | AB psychologie positive OR AB (positive aspect OR positive experience OR positive perceptions OR positive appraisal OR positive impact OR reward OR gain* OR satisfaction* OR benefit* OR uplift* OR meaning* OR enjoyment* OR pleasure OR growth OR hope OR gratification) |  |
| S10 | S8 OR S9 |  |
| S11 | S3 AND S6 AND S10  2855 results for search #11 |  |
| 4. Web of Science | |  |
| #1 | (TS=(dement*)) OR TS=(alzheimer*) |  |
| #2 | (TS=(caregiv*)) OR TS=(carer*) |  |
| #3 | (((((((((((((((TS= (positive aspect)) OR TS= (positive experience)) OR TS= (positive perceptions)) OR TS= (positive appraisal)) OR TS= (positive impact)) OR TS=(reward)) OR TS=(gain*)) OR TS=(satisfaction*)) OR TS=(benefit*)) OR TS=(uplift*)) OR TS=(meaning*)) OR TS=(enjoyment*)) OR TS= (pleasure)) OR TS=(growth)) OR TS=(hope)) OR TS=(gratification)) |  |
| #4 | #1 AND #2 AND #3 |  |
| #5 | (#1 AND #2 AND #3) AND LA=(English)  5085 results for search #6 |  |
| 5. EMBASE | |  |
| #1 | 'dementia'/exp |  |
| #2 | dement*: ab,ti OR alzheimer*:ab,ti |  |
| #3 | #1 OR #2 |  |
| #4 | 'caregiver'/exp |  |
| #5 | caregiv*: ab,ti OR carer*:ab,ti |  |
| #6 | #4 OR #5 |  |
| #7 | 'positive psychology'/exp |  |
| #8 | 'positive aspect'/:ab,ti OR 'positive experience'/:ab,ti OR 'positive perceptions'/:ab,ti OR 'positive appraisal'/:ab,ti OR 'positive impact'/:ab,ti OR reward/:ab,ti OR gain*/:ab,ti OR satisfaction*/:ab,ti OR benefit*/:ab,ti OR uplift*/:ab,ti OR meaning*/:ab,ti OR enjoyment*/:ab,ti OR pleasure/:ab,ti OR growth'/:ab,ti OR hope/:ab,ti OR gratification/:ab,ti |  |
| #9 | #7 OR #8 |  |
| #10 | #3 AND #6 AND #9 |  |
| #11 | English:la |  |
| #12 | #10 AND #11  4889 results for search #12 |  |

**Supplementary S2: Included articles in this review (n=230)**

1. Abdollahpour, I., Nedjat, S., Noroozian, M., Salimi, Y., & Majdzadeh, R. (2017). Positive Aspects of Caregiving Questionnaire: A Validation Study in Caregivers of Patients With Dementia. J Geriatr Psychiatry Neurol, 30(2), 77-83. https://doi.org/10.1177/0891988716686831
2. Abdollahpour, I., Nedjat, S., & Salimi, Y. (2018). Positive Aspects of Caregiving and Caregiver Burden: A Study of Caregivers of Patients With Dementia. J Geriatr Psychiatry Neurol, 31(1), 34-38. https://doi.org/10.1177/0891988717743590
3. Acton, G. J., & Miller, E. W. (2003). Spirituality in caregivers of family members with dementia. J Holist Nurs, 21(2), 117-130. https://doi.org/10.1177/0898010103021002003
4. Alvira, C., Cabrera, E., Kostov, B., Risco, E., Farre, M., Miguel, S., & Zabalegui, A. (2021). Validity and reliability of the Spanish caregiver reaction assessment scale for caregivers of people with dementia. Int J Nurs Pract, 27(4), 9. https://doi.org/10.1111/ijn.12848
5. Alvira, M. C., Risco, E., Cabrera, E., Farre, M., Hallberg, I. R., Bleijlevens, M. H. C., . . . RightTimePlCare, C. (2015). The association between positive-negative reactions of informal caregivers of people with dementia and health outcomes in eight European countries: a cross-sectional study. J Adv Nurs, 71(6), 1417-1434. https://doi.org/10.1111/jan.12528
6. Andren, S., & Elmstahl, S. (2005). Family caregivers' subjective experiences of satisfaction in dementia care: aspects of burden, subjective health and sense of coherence. Scand J Caring Sci, 19(2), 157-168. https://doi.org/10.1111/j.1471-6712.2005.00328.x
7. Ar, Y., & Karanci, A. N. (2019). Turkish Adult Children as Caregivers of Parents with Alzheimer's Disease: Perceptions and Caregiving Experiences. Dementia-International Journal of Social Research and Practice, 18(3), 882-902. https://doi.org/10.1177/1471301217693400
8. Arrigg, A. E. (2015). The relationship between perseverance and finding meaning through caregiving in caregivers of spouses with dementia. (76), ProQuest Information & Learning. Retrieved from https://search.ebscohost.com/login.aspx?direct=true&db=psyh&AN=2015-99180-563&lang=zh-cn&site=ehost-live Available from EBSCOhost APA PsycInfo database.
9. Asiret, G. D., Yilmaz, C. K., & Kasar, K. S. (2021). Investigation of the effects of interventions made according to the Progressively Lowered Stress Threshold Model on the care outcomes of Alzheimer patients and their families: a randomized clinical trial. Psychogeriatrics, 21(5), 738-748. https://doi.org/10.1111/psyg.12734
10. Baker, K. L., Robertson, N., & Connelly, D. (2010). Men caring for wives or partners with dementia: Masculinity, strain and gain. Aging Ment Health, 14(3), 319-327. https://doi.org/10.1080/13607860903228788
11. Bangerter, L. R., Liu, Y., Kim, K., & Zarit, S. H. (2021). Adult day services and dementia caregivers' daily affect: the role of distress response to behavioral and psychological symptoms of dementia. Aging Ment Health, 25(1), 46-52. https://doi.org/10.1080/13607863.2019.1681934
12. Barbabella, F., Poli, A., Andreasson, F., Salzmann, B., Papa, R., Hanson, E., . . . Lamura, G. (2016). A Web-Based Psychosocial Intervention for Family Caregivers of Older People: Results from a Mixed-Methods Study in Three European Countries. JMIR Res Protoc, 5(4), 16. https://doi.org/10.2196/resprot.5847
13. Bar-David, G. N. (1995). The phoenix rising: Self-development in caregivers for relatives with Alzheimer's disease. (55), ProQuest Information & Learning. Retrieved from https://search.ebscohost.com/login.aspx?direct=true&db=psyh&AN=1995-95012-018&lang=zh-cn&site=ehost-live Available from EBSCOhost APA PsycInfo database.
14. Bekhet, A. K. (2013). Effects of positive cognitions and resourcefulness on caregiver burden among caregivers of persons with dementia. International Journal of Mental Health Nursing, 22(4), 340-346. https://doi.org/10.1111/j.1447-0349.2012.00877.x
15. Bekhet, A. K. (2015). Resourcefulness in African American and Caucasian American Caregivers of Persons With Dementia: Associations With Perceived Burden, Depression, Anxiety, Positive Cognitions, and Psychological Well-Being. Perspect Psychiatr Care, 51(4), 285-294. https://doi.org/10.1111/ppc.12095
16. Bekhet, A. K., & Avery, J. S. (2018). Resilience from the perspectives of caregivers of persons with dementia. Arch Psychiatr Nurs, 32(1), 19-23. https://doi.org/10.1016/j.apnu.2017.09.008
17. Bekhet, A. K., & Garnier-Villarreal, M. (2020). Effects of Positive Thinking on Dementia Caregivers' Burden and Care-Recipients' Behavioral Problems. West J Nurs Res, 42(5), 365-372. https://doi.org/10.1177/0193945919861970
18. Berg-Weger, M., Rubio, D. M., & Tebb, S. S. (2000). The caregiver well-being scale revisited. Health Soc Work, 25(4), 255-263. https://doi.org/10.1093/hsw/25.4.255
19. Binford, S. S. (2020). Personalizing care for the caregivers: An exploration of factors contributing to the experiences and outcomes of family caregivers of persons with dementia. (81), ProQuest Information & Learning. Retrieved from https://search.ebscohost.com/login.aspx?direct=true&db=psyh&AN=2020-28122-067&lang=zh-cn&site=ehost-live Available from EBSCOhost APA PsycInfo database.
20. Blume, N. (1999). 'For better or worse': Exploration of positive appraisal in the Alzheimer's caregiving situation. (Ph.D.), University of Kansas. Retrieved from https://potsdam.idm.oclc.org/login?url=https://search.ebscohost.com/login.aspx?direct=true&db=rzh&AN=109874689&site=ehost-live&scope=site Available from EBSCOhost CINAHL Plus with Full Text database.
21. Branger, C., O'Connell, M. E., & Peacock, S. (2018). Protocol for a meta-integration: investigating positive aspects of caregiving in dementia. BMJ Open, 8(7), 8. https://doi.org/10.1136/bmjopen-2017-021215
22. Britton, K., Galioto, R., Tremont, G., Chapman, K., Hogue, O., Carlson, M. D., & Spitznagel, M. B. (2018). Caregiving for a Companion Animal Compared to a Family Member: Burden and Positive Experiences in Caregivers. Front Vet Sci, 5, 10. https://doi.org/10.3389/fvets.2018.00325
23. Butcher, H. K., & Buckwalter, K. C. (2002). Exasperations as blessings: Meaning-making and the caregiving experience. Journal of Aging & Identity, 7(2), 113-132. https://doi.org/10.1023/A:1015439218276
24. Butcher, H. K., Holkup, P. A., & Buckwalter, K. C. (2001). The experience of caring for a family member with Alzheimer's disease. West J Nurs Res, 23(1), 33-55. https://doi.org/10.1177/01939450122044943
25. Carbonneau, H., Caron, C., & Desrosiers, J. (2010). Development of a conceptual framework of positive aspects of caregiving in dementia. Dementia (14713012), 9(3), 327-353. https://doi.org/10.1177/1471301210375316
26. Casey, D., Gallagher, N., Devane, D., Woods, B., Murphy, K., Smyth, S., . . . Doyle, P. (2020). The feasibility of a Comprehensive Resilience-building psychosocial Intervention (CREST) for people with dementia in the community: protocol for a non-randomised feasibility study. Pilot Feasibility Stud, 6(1), 16. https://doi.org/10.1186/s40814-020-00701-2
27. Cheng, S. T., Chan, W. C., Fung, H. H., & Lam, L. C. W. (2021). Self-efficacy in controlling upsetting thoughts, but not positive gains, mediates the effects of benefit-finding group intervention for Alzheimer family caregivers. Psychol Aging. https://doi.org/10.1037/pag0000654
28. Cheng, S. T., Chan, W. C., & Lam, L. C. W. (2019). Long-Term Outcomes of the Benefit-Finding Group Intervention for Alzheimer Family Caregivers: A Cluster-Randomized Double-Blind Controlled Trial. American Journal of Geriatric Psychiatry, 27(9), 984-994. https://doi.org/10.1016/j.jagp.2019.03.013
29. Cheng, S. T., Chan, W. C., & Lam, L. C. W. (2021). Are Care-Recipient Outcomes Attributable to Improved Caregiver Well-Being? A Cluster-Randomized Controlled Trial of Benefit-Finding Intervention. Am J Geriatr Psychiatry. https://doi.org/10.1016/j.jagp.2021.08.009
30. Cheng, S. T., Fung, H. H., Chan, W. C., & Lam, L. C. (2016). Short-Term Effects of a Gain-Focused Reappraisal Intervention for Dementia Caregivers: A Double-Blind Cluster-Randomized Controlled Trial. Am J Geriatr Psychiatry, 24(9), 740-750. https://doi.org/10.1016/j.jagp.2016.04.012
31. Cheng, S. T., Lam, L. C., Kwok, T., Ng, N. S., & Fung, A. W. (2013). Self-efficacy is associated with less burden and more gains from behavioral problems of Alzheimer's disease in Hong Kong Chinese caregivers. Gerontologist, 53(1), 71-80. https://doi.org/10.1093/geront/gns062
32. Cheng, S. T., Lam, L. C. W., Kwok, T., Ng, N. S. S., & Fung, A. W. T. (2013). The Social Networks of Hong Kong Chinese Family Caregivers of Alzheimers Disease: Correlates With Positive Gains and Burden. Gerontologist, 53(6), 998-1008. https://doi.org/10.1093/geront/gns195
33. Cheng, S. T., Lau, R. W. L., Mak, E. P. M., Ng, N. S. S., & Lam, L. C. W. (2014). Benefit-Finding Intervention for Alzheimer Caregivers: Conceptual Framework, Implementation Issues, and Preliminary Efficacy. Gerontologist, 54(6), 1049-1058. https://doi.org/10.1093/geront/gnu018
34. Cheng, S. T., Lau, R. W. L., Mak, E. P. M., Ng, N. S. S., Lam, L. C. W., Fung, H. H., . . . Lee, D. T. F. (2012). A benefit-finding intervention for family caregivers of persons with Alzheimer disease: study protocol of a randomized controlled trial. Trials, 13, 10. https://doi.org/10.1186/1745-6215-13-98
35. Cheng, S. T., Mak, E. P. M., Fung, H. H., Kwok, T., Lee, D. T. F., & Lam, L. C. W. (2017). Benefit-Finding and Effect on Caregiver Depression: A Double-Blind Randomized Controlled Trial. J Consult Clin Psychol, 85(5), 521-529. https://doi.org/10.1037/ccp0000176
36. Cheng, S. T., Mak, E. P. M., Kwok, T., Fung, H., & Lam, L. C. W. (2020). Benefit-Finding Intervention Delivered Individually to Alzheimer Family Caregivers: Longer-Term Outcomes of a Randomized Double-Blind Controlled Trial. Journals of Gerontology Series B-Psychological Sciences and Social Sciences, 75(9), 1884-1893. https://doi.org/10.1093/geronb/gbz118
37. Cheng, S. T., Mak, E. P. M., Lau, R. W. L., Ng, N. S. S., & Lam, L. C. W. (2016). Voices of Alzheimer Caregivers on Positive Aspects of Caregiving. Gerontologist, 56(3), 451-460. https://doi.org/10.1093/geront/gnu118
38. Cherry, M. G., Ablett, J. R., Dickson, J. M., Powell, D., Sikdar, S., & Salmon, P. (2019). A qualitative study of the processes by which carers of people with dementia derive meaning from caring. Aging Ment Health, 23(1), 69-76. https://doi.org/10.1080/13607863.2017.1393792
39. Cho, J. M., Ory, M. G., & Stevens, A. B. (2016). Socioecological factors and positive aspects of caregiving: findings from the REACH II intervention. Aging Ment Health, 20(11), 1190-1201. https://doi.org/10.1080/13607863.2015.1068739
40. Cohen, C. A., Colantonio, A., & Vernich, L. (2002). Positive aspects of caregiving: rounding out the caregiver experience. Int J Geriatr Psychiatry, 17(2), 184-188. https://doi.org/10.1002/gps.561
41. Cohen, C. A., Gold, D. P., Shulman, K. I., & Zucchero, C. A. (1994). Positive aspects in caregiving: An overlooked variable in research. Canadian Journal on Aging, 13(3), 378-391. https://doi.org/10.1017/S071498080000619X
42. Cohen, H. L., & Lee, Y. (2006). Dementia caregivers: rewards in multicultural perspectives. Journal of Human Behavior in the Social Environment, 14(1/2), 299-324.
43. Corbeil, R. R., Quayhagen, M. P., & Quayhagen, M. (1999). Intervention effects on dementia caregiving interaction A stress-adaptation modeling approach. J Aging Health, 11(1), 79-95. https://doi.org/10.1177/089826439901100105
44. de Labra, C., Millan-Calenti, J. C., Bujan, A., Nunez-Naveira, L., Jensen, A. M., Peersen, M. C., . . . Maseda, A. (2015). Predictors of caregiving satisfaction in informal caregivers of people with dementia. Arch Gerontol Geriatr, 60(3), 380-388. https://doi.org/10.1016/j.archger.2015.03.002
45. DeGregory, C. (2014). The effects of multiple gratitude interventions among informal caregivers of persons with dementia and Alzheimer's disease. (Ph.D.), University of South Carolina. Retrieved from https://potsdam.idm.oclc.org/login?url=https://search.ebscohost.com/login.aspx?direct=true&db=rzh&AN=109786418&site=ehost-live&scope=site Available from EBSCOhost CINAHL Plus with Full Text database.
46. Devi, F., Yuan, Q., Wang, P. Z., Tan, G. T. H., Goveas, R. R., Ng, L. L., . . . Subramaniam, M. (2020). Positive aspect of caregiving among primary informal dementia caregivers in Singapore. PLoS One, 15(8), 11. https://doi.org/10.1371/journal.pone.0237677
47. Dias, R., Santos, R. L., Sousa, M. F., Nogueira, M. M., Torres, B., Belfort, T., & Dourado, M. C. (2015). Resilience of caregivers of people with dementia: a systematic review of biological and psychosocial determinants. Trends Psychiatry Psychother, 37(1), 12-19. https://doi.org/10.1590/2237-6089-2014-0032
48. DiBartolo, M. C. (2002). Exploring self-efficacy and hardiness in spousal caregivers of individuals with dementia. J Gerontol Nurs, [28](4), 24-33.
49. Dieker, J. (2021). Meaning-focused coping in dementia caregiving: Predicting positive and negative outcomes. (82), ProQuest Information & Learning. Retrieved from https://search.ebscohost.com/login.aspx?direct=true&db=psyh&AN=2020-86254-297&lang=zh-cn&site=ehost-live Available from EBSCOhost APA PsycInfo database.
50. Donnellan, W. J., Bennett, K. M., & Watson, N. (2021). Exploring resilience in adult daughter and spousal carers of people living with dementia in North West England: an ecological approach. Quality in Ageing and Older Adults, 22(1), 40-55. https://doi.org/10.1108/qaoa-12-2020-0063
51. Donovan, M. L., & Corcoran, M. A. (2010). Description of Dementia Caregiver Uplifts and Implications for Occupational Therapy. American Journal of Occupational Therapy, 64(4), 590-595. https://doi.org/10.5014/ajot.2010.09064
52. Doyle, P., Gallagher, N., Smyth, S., & Casey, D. (2020). Exploring the feasibility and acceptability of a Comprehensive Resilience-building psychosocial intervention (CREST) for people with dementia in the community: A nonrandomised feasibility study. Int Psychogeriatr, 32(SUPPL 1), 101. https://doi.org/10.1017/S1041610220002410
53. Duggleby, W., Ploeg, J., McAiney, C., Fisher, K., Ruiz, K. J., Ghosh, S., . . . Swindle, J. (2019). A Comparison of Users and Nonusers of a Web-Based Intervention for Carers of Older Persons With Alzheimer Disease and Related Dementias: Mixed Methods Secondary Analysis. J Med Internet Res, 21(10), 13. https://doi.org/10.2196/14254
54. Duggleby, W., Ploeg, J., McAiney, C., Peacock, S., Fisher, K., Ghosh, S., . . . Ruiz, K. J. (2018). Web-Based Intervention for Family Carers of Persons with Dementia and Multiple Chronic Conditions (My Tools 4 Care): Pragmatic Randomized Controlled Trial. J Med Internet Res, 20(6), 14. https://doi.org/10.2196/10484
55. Duggleby, W., Williams, A., Wright, K., & Bollinger, S. (2009). Renewing everyday hope: the hope experience of family caregivers of persons with dementia. Issues Ment Health Nurs, 30(8), 514-521. https://doi.org/10.1080/01612840802641727
56. Duran-Gomez, N., Guerrero-Martin, J., Perez-Civantos, D., Jurado, C. F. L., Palonno-Looez, P., & Caceres, M. C. (2020). Understanding Resilience Factors Among Caregivers of People with Alzheimer's Disease in Spain. Psychol Res Behav Manag, 13, 1011-1025. https://doi.org/10.2147/prbm.S274758
57. Faba, J., Villar, F., & Giuliani, M. F. (2017). Development of a measure to evaluate gains among spanish dementia caregivers: The gains associated with caregiving (GAC) scale. Arch Gerontol Geriatr, 68, 76-83. https://doi.org/10.1016/j.archger.2016.09.004
58. Farran, C. J. (1997). Theoretical perspectives concerning positive aspects of caring for elderly persons with dementia: stress/adaptation with existentialism. Gerontologist, 37(2), 250-256. https://doi.org/10.1093/geront/37.2.250
59. Farran, C. J., Keane-Hagerty, E., Salloway, S., Kupferer, S., & Wilken, C. S. (1991). Finding meaning: an alternative paradigm for Alzheimer's disease family caregivers. Gerontologist, 31(4), 483-489. https://doi.org/10.1093/geront/31.4.483
60. Farran, C. J., & Kuhn, D. R. (1998). Finding meaning through caring for persons with Alzheimer's disease: Assessment and intervention. In P. T. P. Wong & P. S. Fry (Eds.), The human quest for meaning: A handbook of psychological research and clinical applications. (pp. 335-358). Mahwah, NJ: Lawrence Erlbaum Associates Publishers.
61. Farran, C. J., Miller, B. H., Kaufman, J. E., & Davis, L. (1997). Race, finding meaning, and caregiver distress. J Aging Health, 9(3), 316-333. https://doi.org/10.1177/089826439700900303
62. Farran, C. J., Miller, B. H., Kaufman, J. E., Donner, E., & Fogg, L. (1999). Finding meaning through caregiving: Development of an instrument for family caregivers of persons with Alzheimer's disease. J Clin Psychol, 55(9), 1107-1125. https://doi.org/10.1002/(sici)1097-4679(199909)55:9<1107::Aid-jclp8>3.0.Co;2-v
63. Fernández-Calvo, B., Castillo, I. C., Campos, F. R., Carvalho, V. M. d. L. e., Silva, J. C. d., & Torro-Alves, N. (2016). Resilience in caregivers of persons with Alzheimer’s disease: A human condition to overcome caregiver vulnerability. Estudos de Psicologia, 21. https://doi.org/10.5935/1678-4669.20160013
64. Fields, N. L., Xu, L., & Miller, V. J. (2019). Caregiver Burden and Desire for Institutional Placement-The Roles of Positive Aspects of Caregiving and Religious Coping. American Journal of Alzheimers Disease and Other Dementias, 34(3), 199-207. https://doi.org/10.1177/1533317519826217
65. Franks, M. M., & Stephens, M. A. P. (1996). Social support in the context of caregiving: Husbands' provision of support to wives involved in parent care. Journals of Gerontology Series B-Psychological Sciences and Social Sciences, 51(1), P43-P52. https://doi.org/10.1093/geronb/51B.1.P43
66. Fuju, T., Yamagami, T., Yamaguchi, H., & Yamazaki, T. (2021). Development of the Dementia Caregiver Positive Feeling Scale 21‐item version (DCPFS‐21) in Japan to recognise positive feelings about caregiving for people with dementia. Psychogeriatrics, 21(4), 650-658. <https://doi.org/10.1111/psyg.12727>
67. Fuju, T., Yamagami, T., Yamaguchi, H., & Yamazaki, T. (2021). A randomized controlled trial of the "positive diary" intervention for family caregivers of people with dementia. Perspect Psychiatr Care. https://doi.org/10.1111/ppc.13013
68. Furukawa, H., & Greiner, C. (2021). Reliability and validation of the Positive Aspects of Caregiving scale among Japanese caregivers of people with dementia. International Journal of Nursing Sciences, 8(2), 210-214. https://doi.org/10.1016/j.ijnss.2021.03.007
69. Garcia-Castro, F. J., Alba, A., & Blanca, M. J. (2021). The role of character strengths in predicting gains in informal caregivers of dementia. Aging Ment Health, 25(1), 32-37. https://doi.org/10.1080/13607863.2019.1667298
70. Garcia-Castro, F. J., Hernandez, A., & Blanca, M. J. (2021). Life satisfaction and the mediating role of character strengths and gains in informal caregivers. J Psychiatr Ment Health Nurs, 13. https://doi.org/10.1111/jpm.12764
71. García-Castro, F. J., Holgado-Tello, F. P., & Blanca, M. J. (2021). New Evidence for the Psychometric Properties of the Spanish Version of the Gain in Alzheimer Care Instrument. Span J Psychol, 24, e25. https://doi.org/10.1017/sjp.2021.32
72. Garcia-Mochon, L., Pena-Longobardo, L. M., del Rio-Lozano, M., Oliva-Moreno, J., Larranaga-Padilla, I., & Garcia-Calvente, M. D. (2019). Determinants of Burden and Satisfaction in Informal Caregivers: Two Sides of the Same Coin? The CUIDAR-SE Study. Int J Environ Res Public Health, 16(22), 13. https://doi.org/10.3390/ijerph16224378
73. George, C., & Ferreira, N. (2020). Carer burden and positive gain in dementia. Working with Older People: Community Care Policy & Practice, 24(2), 113-123. https://doi.org/10.1108/WWOP-02-2020-0009
74. Gitlin, L. N., Winter, L., Dennis, M. P., & Hauck, W. W. (2006). Assessing perceived change in the well-being of family caregivers: psychometric properties of the Perceived Change Index and response patterns. American Journal of Alzheimer's Disease & Other Dementias, 21(5), 304-311. https://doi.org/10.1177/1533317506292283
75. Gold, D. P., Cohen, C., Shulman, K., Zucchero, C., Andres, D., & Etezadi, J. (1995). Caregiving and dementia: Predicting negative and positive outcomes for caregivers. Int J Aging Hum Dev, 41(3), 183-201. https://doi.org/10.2190/rgyj-5ka2-7thx-7bq5
76. Goncalves-Pereira, M., Carmo, I., da Silva, J. A., Papoila, A. L., Mateos, R., & Zarit, S. H. (2010). Caregiving experiences and knowledge about dementia in Portuguese clinical outpatient settings. Int Psychogeriatr, 22(2), 270-280. https://doi.org/10.1017/s1041610209991050
77. Goncalves-Pereira, M., Zarit, S. H., Cardoso, A. M., Alves da Silva, J., Papoila, A. L., & Mateos, R. (2020). A comparison of primary and secondary caregivers of persons with dementia. Psychol Aging, 35(1), 20-27. https://doi.org/10.1037/pag0000380
78. Goncalves-Pereira, M., Zarit, S. H., Papoila, A. L., & Mateos, R. (2021). Positive and negative experiences of caregiving in dementia: The role of sense of coherence. Int J Geriatr Psychiatry, 36(2), 360-367. https://doi.org/10.1002/gps.5433
79. Goode, K. T., Haley, W. E., Roth, D. L., & Ford, G. R. (1998). Predicting longitudinal changes in caregiver physical and mental health: A stress process model. Health Psychology, 17(2), 190-198. https://doi.org/10.1037/0278-6133.17.2.190
80. Gottlieb, B. H., & Rooney, J. A. (2004). Coping effectiveness: determinants and relevance to the mental health and affect of family caregivers of persons with dementia. Aging Ment Health, 8(4), 364-373. https://doi.org/10.1080/13607860410001709719
81. Grover, S., Nehra, R., Malhotra, R., & Kate, N. (2017). Positive Aspects of Caregiving Experience among Caregivers of Patients with Dementia. East Asian Arch Psychiatry, 27(2), 71-78.
82. Haley, W. E., Brown, S. L., & Levine, E. G. (1987). Experimental evaluation of the effectiveness of group intervention for dementia caregivers. Gerontologist, 27(3), 376-382. https://doi.org/10.1093/geront/27.3.376
83. Han, A. (2020). Interventions for Attitudes and Empathy Toward People With Dementia and Positive Aspects of Caregiving: A Systematic Review and Meta-Analysis. Res Aging, 42(2), 72-82. https://doi.org/10.1177/0164027519884766
84. Harmell, A. L., Mausbach, B. T., Roepke, S. K., Moore, R. C., von Kanel, R., Patterson, T. L., . . . Grant, I. (2011). The relationship between self-efficacy and resting blood pressure in spousal Alzheimer's caregivers. Br J Health Psychol, 16, 317-328. https://doi.org/10.1348/135910710x504932
85. Harwood, D. G., Barker, W. W., Ownby, R. L., Bravo, M., Aguero, H., & Duara, R. (2000). Predictors of positive and negative appraisal among Cuban American caregivers of Alzheimer's disase patients. Int J Geriatr Psychiatry, 15(6), 481-487. https://doi.org/10.1002/1099-1166(200006)15:6<481::AID-GPS984>3.0.CO;2-J
86. Heo, G. J. (2014). Religious Coping, Positive Aspects of Caregiving, and Social Support Among Alzheimer's Disease Caregivers. Clin Gerontol, 37(4), 368-385. https://doi.org/10.1080/07317115.2014.907588
87. Hilgeman, M. M., Allen, R. S., DeCoster, J., & Burgio, L. D. (2007). Positive aspects of caregiving as a moderator of treatment outcome over 12 months. Psychol Aging, 22(2), 361-371. https://doi.org/10.1037/0882-7974.22.2.361
88. Hodge, D. R., & Sun, F. (2012). Positive feelings of caregiving among Latino Alzheimer's family caregivers: Understanding the role of spirituality. Aging Ment Health, 16(6), 689-698. https://doi.org/10.1080/13607863.2012.678481
89. Irvin, B. L. (1993). Social support, self-worth and hope as self-care resources for coping with caregiver stress. (PH.D.), UNIVERSITY OF TEXAS AT AUSTIN. Retrieved from https://potsdam.idm.oclc.org/login?url=https://search.ebscohost.com/login.aspx?direct=true&db=rzh&AN=109871755&site=ehost-live&scope=site Available from EBSCOhost CINAHL Plus with Full Text database.
90. Irvin, B. L., & Acton, G. J. (1997). Stress, hope, and well-being of women caring for family members with Alzheimer's disease. Holist Nurs Pract, 11(2), 69-79. https://doi.org/10.1097/00004650-199701000-00010
91. Jansen, A. P., van Hout, H. P., van Marwijk, H. W., Nijpels, G., Gundy, C., Vernooij-Dassen, M. J., . . . Stalman, W. A. (2007). Sense of competence questionnaire among informal caregivers of older adults with dementia symptoms: a psychometric evaluation. Clin Pract Epidemiol Ment Health, 3, 11. https://doi.org/10.1186/1745-0179-3-11
92. Johansson, M. F., McKee, K. J., Dahlberg, L., Summer Meranius, M., Williams, C. L., & Marmstål Hammar, L. (2022). Negative Impact and Positive Value of Caregiving in Spouse Carers of Persons with Dementia in Sweden. Int J Environ Res Public Health, 19(3). https://doi.org/10.3390/ijerph19031788
93. Joling, K. J., Windle, G., Droes, R. M., Huisman, M., Hertogh, C., & Woods, R. T. (2017). What are the essential features of resilience for informal caregivers of people living with dementia? A Delphi consensus examination. Aging Ment Health, 21(5), 509-517. https://doi.org/10.1080/13607863.2015.1124836
94. Jones, S. M., Killett, A., & Mioshi, E. (2019). The role of resilient coping in dementia carers' wellbeing. British Journal of Neuroscience Nursing, 15(1), 6-12. https://doi.org/10.12968/bjnn.2019.15.1.6
95. Kajiwara, K., Nakatani, H., Ono, M., & Miyakoshi, Y. (2015). Positive appraisal of in‐home family caregivers of dementia patients as an influence on the continuation of caregiving. Psychogeriatrics, 15(1), 26-31. https://doi.org/10.1111/psyg.12074
96. Kalaitzaki, A., Koukouli, S., Foukaki, M. E., Markakis, G., & Tziraki, C. (2021). Dementia Family Carers' Quality of Life and Their Perceptions About Care-receivers' Dementia Symptoms: The Role of Resilience. J Aging Health, 10. https://doi.org/10.1177/08982643211050206
97. Kim, D., Kwon, J. A., & Han, E. K. (2019). Integrative literature review on resilience of family with the elderly dementia. Indian Journal of Public Health Research and Development, 10(11), 4598-4602. https://doi.org/10.5958/0976-5506.2019.04332.8
98. Kim, G. M., Lim, H. S., Lim, J. Y., Kim, S. S., & Kim, E. J. (2019). Effects of a Family Resilience Enhancement Program (FREP) on Family Adaptation to Elderly with Dementia in South Korea. International Journal of Gerontology, 13(1), 54-58. https://doi.org/10.6890/ijge.201903_13(1).0011
99. Kinney, J. M., & Stephens, M. A. (1989). Hassles and uplifts of giving care to a family member with dementia. Psychol Aging, 4(4), 402-408. https://doi.org/10.1037//0882-7974.4.4.402
100. Ko, J. W. (2011). Alzheimer's disease and related disorders caregiver's acceptance of a web-based structured written emotional expression intervention. (Ph.D.), University of Iowa. Retrieved from https://potsdam.idm.oclc.org/login?url=https://search.ebscohost.com/login.aspx?direct=true&db=rzh&AN=109858571&site=ehost-live&scope=site Available from EBSCOhost CINAHL Plus with Full Text database.
101. Kobiske, K. R., & Bekhet, A. K. (2018). Resilience in Caregivers of Partners With Young Onset Dementia: A Concept Analysis. Issues Ment Health Nurs, 39(5), 411-419. https://doi.org/10.1080/01612840.2017.1400625
102. Koerner, S. S., Kenyon, D. B., & Shirai, Y. (2009). Caregiving for elder relatives: Which caregivers experience personal benefits/gains? Arch Gerontol Geriatr, 48(2), 238-245. https://doi.org/10.1016/j.archger.2008.01.015
103. Kramer, B. J. (1993). MARITAL HISTORY AND THE PRIOR RELATIONSHIP AS PREDICTORS OF POSITIVE AND NEGATIVE OUTCOMES AMONG WIFE CAREGIVERS. Family Relations, 42(4), 367-375. https://doi.org/10.2307/585336
104. Lamont, R. A., Quinn, C., Nelis, S. M., Martyr, A., Rusted, J. M., Hindle, J. V., . . . Team, I. S. (2019). Self-esteem, self-efficacy, and optimism as psychological resources among caregivers of people with dementia: findings from the IDEAL study. Int Psychogeriatr, 31(9), 1259-1266. https://doi.org/10.1017/s1041610219001236
105. Lau, W. Y. T., Stoner, C., Wong, G. H. Y., & Spector, A. (2021). New horizons in understanding the experience of Chinese people living with dementia: a positive psychology approach. Age Ageing, 50(5), 1493-1498. https://doi.org/10.1093/ageing/afab097
106. Lawton, M. P., Kleban, M. H., Moss, M., Rovine, M., & Glicksman, A. (1989). Measuring caregiving appraisal. J Gerontol, 44(3), P61-71. https://doi.org/10.1093/geronj/44.3.p61
107. Lawton, M. P., Moss, M., Kleban, M. H., Glicksman, A., & Rovine, M. (1991). A two-factor model of caregiving appraisal and psychological well-being. J Gerontol, 46(4), P181-189. https://doi.org/10.1093/geronj/46.4.p181
108. Lawton, M. P., Rajagopal, D., Brody, E., & Kleban, M. H. (1992). The dynamics of caregiving for a demented elder among black and white families. J Gerontol, 47(4), S156-164. <https://doi.org/10.1093/geronj/47.4.s156>
109. Lee, E. E., Farran, C. J., Tripp-Reimer, T., & Sadler, G. R. (2003). Assessing the cultural appropriateness of the Finding Meaning Through Caregiving Scale for Korean caregivers. J Nurs Meas, 11(1), 19-28. https://doi.org/10.1891/jnum.11.1.19.52060
110. Lee, M. J. (2006). The effects of reach interventions on longitudinal changes of negative and positive caregiver outcomes. (67), ProQuest Information & Learning. Retrieved from https://search.ebscohost.com/login.aspx?direct=true&db=psyh&AN=2006-99015-082&lang=zh-cn&site=ehost-live Available from EBSCOhost APA PsycInfo database.
111. Lee, Y., & Bronstein, L. R. (2010). When do Korean-American dementia caregivers find meaning in caregiving?: The role of culture and differences between spouse and child caregivers. Journal of Ethnic & Cultural Diversity in Social Work: Innovation in Theory, Research & Practice, 19(1), 73-86. https://doi.org/10.1080/15313200903547756
112. Lee, Y., & Choi, S. (2013). Korean American dementia caregivers' attitudes toward caregiving: the role of social network versus satisfaction with social support. J Appl Gerontol, 32(4), 422-442. https://doi.org/10.1177/0733464811431163
113. Leggett, A. N., Meyer, O. L., Bugajski, B. C., & Polenick, C. A. (2021). Accentuate the Positive: The Association Between Informal and Formal Supports and Caregiving Gains. Journal of Applied Gerontology, 40(7), 763-771. https://doi.org/10.1177/0733464820914481
114. Leipold, B., Schacke, C., & Zank, S. (2008). Personal growth and cognitive complexity in caregivers of patients with dementia. Eur J Ageing, 5(3), 203-214. https://doi.org/10.1007/s10433-008-0090-8
115. Lethin, C., Renom-Guiteras, A., Zwakhalen, S., Soto-Martin, M., Saks, K., Zabalegui, A., . . . Karlsson, S. (2017). Psychological well-being over time among informal caregivers caring for persons with dementia living at home. Aging Ment Health, 21(11), 1138-1146. https://doi.org/10.1080/13607863.2016.1211621
116. Levesque, L., Cossette, S., & Lachance, L. (1998). Predictors of the psychological well-being of primary caregivers living with a demented relative: A 1-year follow-up study. Journal of Applied Gerontology, 17(2), 240-258. https://doi.org/10.1177/073346489801700211
117. Levesque, L., Cossette, S., & Laurin, L. (1995). A Multidimensional Examination of the Psychological and Social Well-Being of Caregivers of A Demented Relative. Res Aging, 17(3), 332-360. https://doi.org/10.1177/0164027595173005
118. Liew, T. M., Luo, N., Ng, W. Y., Chionh, H. L., Goh, J., & Yap, P. (2010). Predicting Gains in Dementia Caregiving. Dement Geriatr Cogn Disord, 29(2), 115-122. https://doi.org/10.1159/000275569
119. Liew, T. M., Tai, B. C., Wee, S. L., Koh, G. C. H., & Yap, P. (2020). The Longitudinal Effects of Caregiver Grief in Dementia and the Modifying Effects of Social Services: A Prospective Cohort Study. J Am Geriatr Soc, 68(10), 2348-2353. https://doi.org/10.1111/jgs.16717
120. Lim, J., Griva, K., Goh, J., Chionh, H. L., & Yap, P. (2011). Coping Strategies Influence Caregiver Outcomes Among Asian Family Caregivers of Persons With Dementia in Singapore. Alzheimer Disease & Associated Disorders, 25(1), 34-41. https://doi.org/10.1097/WAD.0b013e3181ec18ae
121. Lindeza, P., Rodrigues, M., Costa, J., Guerreiro, M., & Rosa, M. M. (2020). Impact of dementia on informal care: a systematic review of family caregivers' perceptions. BMJ Support Palliat Care. https://doi.org/10.1136/bmjspcare-2020-002242
122. Liu, C., Marino, V. R., Howard, V. J., Haley, W. E., & Roth, D. L. (2021). Positive aspects of caregiving in incident and long-term caregivers: Role of social engagement and distress. Aging Ment Health, 7. https://doi.org/10.1080/13607863.2021.2000935
123. Liu, H. Y., & Huang, L. H. (2018). The relationship between family functioning and caregiving appraisal of dementia family caregivers: caregiving self-efficacy as a mediator. Aging Ment Health, 22(4), 558-567. https://doi.org/10.1080/13607863.2016.1269148
124. Liu, J. Y., Lou, Y. F., Wu, B., & Mui, A. (2021). "I've been always strong to conquer any suffering:" challenges and resilience of Chinese American dementia caregivers in a life course perspective. Aging Ment Health, 25(9), 1716-1724. https://doi.org/10.1080/13607863.2020.1793900
125. Liu, W. (2009). Examining the effects of positive aspects of caregiving, mood, and social support in dementia caregivers. (69), ProQuest Information & Learning. Retrieved from https://search.ebscohost.com/login.aspx?direct=true&db=psyh&AN=2009-99040-382&lang=zh-cn&site=ehost-live Available from EBSCOhost APA PsycInfo database.
126. Liu, Y., Insel, K. C., Reed, P. G., & Crist, J. D. (2012). Family Caregiving of Older Chinese People With Dementia Testing a Model. Nurs Res, 61(1), 39-50. https://doi.org/10.1097/NNR.0b013e31823bc451
127. Lloyd, J., Patterson, T., & Muers, J. (2016). The positive aspects of caregiving in dementia: A critical review of the qualitative literature. Dementia-International Journal of Social Research and Practice, 15(6), 1534-1561. https://doi.org/10.1177/1471301214564792
128. Lloyd, S. M. (2008). Spirituality, meaning making, and grief in Alzheimer's caregivers. (68), ProQuest Information & Learning. Retrieved from https://search.ebscohost.com/login.aspx?direct=true&db=psyh&AN=2008-99080-530&lang=zh-cn&site=ehost-live Available from EBSCOhost APA PsycInfo database.
129. Lopez, J., Romero-Moreno, R., Marquez-González, M., & Losada, A. (2012). Spirituality and self-efficacy in dementia family caregiving: Trust in God and yourself. Int Psychogeriatr, 24(12), 1943-1952. https://doi.org/10.1017/S1041610212001287
130. López, J., Romero-Moreno, R., Márquez-González, M., & Losada, A. (2015). Anger and health in dementia caregivers: exploring the mediation effect of optimism. Stress and Health, 31(2), 158-165. https://doi.org/10.1002/smi.2539
131. Lou, V. W. Q., Lau, B. H. P., & Cheung, K. S. L. (2015). Positive aspects of caregiving (PAC): Scale validation among Chinese dementia caregivers (CG). Arch Gerontol Geriatr, 60(2), 299-306. https://doi.org/10.1016/j.archger.2014.10.019
132. Mausbach, B. T., Roepke, S. K., Depp, C. A., Patterson, T. L., & Grant, I. (2009). Specificity of cognitive and behavioral variables to Positive and Negative Affect. Behav Res Ther, 47(7), 608-615. https://doi.org/10.1016/j.brat.2009.04.006
133. Mayor, M. S., Ribeiro, O., & Paul, C. (2009). Satisfaction In Dementia and Stroke Caregivers: A Comparative Study. Rev Lat Am Enfermagem, 17(5), 620-624. https://doi.org/10.1590/s0104-11692009000500004
134. Mbiza, S. T. (2016). Ethnic differences in the impact of psychosocial resources on well-being of family caregivers of individuals with dementia. (76), ProQuest Information & Learning. Retrieved from https://search.ebscohost.com/login.aspx?direct=true&db=psyh&AN=2016-16233-080&lang=zh-cn&site=ehost-live Available from EBSCOhost APA PsycInfo database.
135. McAuliffe, L., Ong, B., & Kinsella, G. (2020). Mediators of burden and depression in dementia family caregivers: Kinship differences. Dementia-International Journal of Social Research and Practice, 19(7), 2234-2250. https://doi.org/10.1177/1471301218819345
136. McLennon, S. M. (2008). The physical and mental health of spouse caregivers in dementia: finding meaning as a mediator of burden. (Ph.D.), University of Alabama at Birmingham. Retrieved from https://potsdam.idm.oclc.org/login?url=https://search.ebscohost.com/login.aspx?direct=true&db=rzh&AN=109850423&site=ehost-live&scope=site Available from EBSCOhost CINAHL Plus with Full Text database.
137. McLennon, S. M., Habermann, B., & Rice, M. (2011). Finding meaning as a mediator of burden on the health of caregivers of spouses with dementia. Aging Ment Health, 15(4), 522-530. https://doi.org/10.1080/13607863.2010.543656
138. Merlo, P., Devita, M., Mandelli, A., Rusconi, M. L., Taddeucci, R., Terzi, A., . . . Mondini, S. (2018). Alzheimer Cafe: an approach focused on Alzheimer's patients but with remarkable values on the quality of life of their caregivers. Aging Clin Exp Res, 30(7), 767-774. https://doi.org/10.1007/s40520-017-0844-2
139. Merrilees, J. J., Bernstein, A., Dulaney, S., Heunis, J., Walker, R., Rah, E., . . . Bonasera, S. J. (2020). The Care Ecosystem: Promoting self-efficacy among dementia family caregivers. Dementia-International Journal of Social Research and Practice, 19(6), 1955-1973. https://doi.org/10.1177/1471301218814121
140. Metcalfe, A., Jones, B., Mayer, J., Gage, H., Oyebode, J., Boucault, S., . . . Kurz, A. (2019). Online information and support for carers of people with young-onset dementia: A multi-site randomised controlled pilot study. Int J Geriatr Psychiatry, 34(10), 1455-1464. https://doi.org/10.1002/gps.5154
141. Monin, J. K., Schulz, R., & Feeney, B. C. (2015). Compassionate Love in Individuals With Alzheimer's Disease and Their Spousal Caregivers: Associations With Caregivers' Psychological Health. Gerontologist, 55(6), 981-989. https://doi.org/10.1093/geront/gnu001
142. Monteiro, A. M. F., Neto, J. P. S., Santos, R. L., Kimura, N., Baptista, M. A. T., & Dourado, M. C. N. (2021). Factor analysis of the Resilience Scale for Brazilian caregivers of people with Alzheimer's disease. Trends Psychiatry Psychother, 43(4), 311-319. https://doi.org/10.47626/2237-6089-2020-0179
143. Morano, C. L. (2000). Comparative analysis of a moderating and mediating model of stress, appraisal, and coping in Hispanic and non-Hispanic Alzheimer's disease caregivers. (60), ProQuest Information & Learning. Retrieved from https://search.ebscohost.com/login.aspx?direct=true&db=psyh&AN=2000-95009-067&lang=zh-cn&site=ehost-live Available from EBSCOhost APA PsycInfo database.
144. Morano, C. L. (2003). The Role of Appraisal and Expressive Support in Mediating Strain and Gain in Hispanic Alzheimer's Disease Caregivers. Journal of Ethnic & Cultural Diversity in Social Work: Innovation in Theory, Research & Practice, 12(2), 1-18. https://doi.org/10.1300/J051v12n02_01
145. Morimoto, H., & Takebayashi, Y. (2021). Antecedents and Outcomes of Enrichment Among Working Family Caregivers of People With Dementia: A Longitudinal Analysis. Journals of Gerontology Series B-Psychological Sciences and Social Sciences, 76(6), 1060-1070. https://doi.org/10.1093/geronb/gbaa183
146. Moskowitz, J. T., Cheung, E. O., Snowberg, K. E., Verstaen, A., Merrilees, J., Salsman, J. M., & Dowling, G. A. (2019). Randomized Controlled Trial of a Facilitated Online Positive Emotion Regulation Intervention for Dementia Caregivers. Health Psychology, 38(5), 391-402. https://doi.org/10.1037/hea0000680
147. Motenko, A. K. (1989). The Frustrations, Gratifications, and Well-Being of Dementia Caregivers1. Gerontologist, 29(2), 166-172.
148. Murphy, M. R. (2006). Positive aspects of family caregiving of patients with Alzheimer's disease: A qualitative study. (66), ProQuest Information & Learning. Retrieved from https://search.ebscohost.com/login.aspx?direct=true&db=psyh&AN=2006-99004-091&lang=zh-cn&site=ehost-live Available from EBSCOhost APA PsycInfo database.
149. Murray, J., Schneider, J., Banerjee, S., & Mann, A. (1999). EUROCARE: a cross-national study of co-resident spouse carers for people with Alzheimer's disease: II--A qualitative analysis of the experience of caregiving. Int J Geriatr Psychiatry, 14(8), 662-667. https://doi.org/10.1002/(sici)1099-1166(199908)14:8<662::aid-gps993>3.0.co;2-4
150. Narayan, S., Lewis, M., Tornatore, J., Hepburn, K., & Corcoran-Perry, S. (2001). Subjective responses to caregiving for a spouse with dementia. J Gerontol Nurs, 27(3), 19-28. https://doi.org/10.3928/0098-9134-20010301-05
151. Netto, N. R., Jenny, G. Y. N., & Philip, Y. L. K. (2009). Growing and gaining through caring for a loved one with dementia. Dementia (14713012), 8(2), 245-261. https://doi.org/10.1177/1471301209103269
152. O'Rourke, N., Kupferschmidt, A. L., Claxton, A., Smith, J. Z., Chappell, N., & Beattie, B. L. (2010). Psychological resilience predicts depressive symptoms among spouses of persons with Alzheimer disease over time. Aging Ment Health, 14(8), 984-993. https://doi.org/10.1080/13607863.2010.501063
153. Pallant, J. F., & Reid, C. (2014). Measuring the positive and negative aspects of the caring role in community versus aged care setting. Australas J Ageing, 33(4), 244-249. https://doi.org/10.1111/ajag.12046
154. Pang, C. K. (2019). Finding meaning in Chinese dementia family caregiving: A grounded theory study. (80), ProQuest Information & Learning. Retrieved from https://search.ebscohost.com/login.aspx?direct=true&db=psyh&AN=2019-00353-035&lang=zh-cn&site=ehost-live Available from EBSCOhost APA PsycInfo database.
155. Pankong, O., Pothiban, L., Sucamvang, K., & Khampolsiri, T. (2018). A Randomized Controlled Trial of Enhancing Positive Aspects of Caregiving in Thai Dementia Caregivers for Dementia. Pacific Rim International Journal of Nursing Research, 22(2), 131-143.
156. Paul, C., Teixeira, L., Duarte, N., Pires, C. L., & Ribeiro, O. (2019). Effects of a community intervention program for dementia on mental health: the importance of secondary caregivers in promoting positive aspects and reducing strain. Community Ment Health J, 55(2), 296-303. https://doi.org/10.1007/s10597-018-0345-6
157. Peacock, S., Forbes, D., Markle-Reid, M., Hawranik, P., Morgan, D., Jansen, L., . . . Henderson, S. R. (2010). The Positive Aspects of the Caregiving Journey With Dementia: Using a Strengths-Based Perspective to Reveal Opportunities. Journal of Applied Gerontology, 29(5), 640-659. https://doi.org/10.1177/0733464809341471
158. Pertl, M. M., Sooknarine-Rajpatty, A., Brennan, S., Robertson, I. H., & Lawlor, B. A. (2019). Caregiver Choice and Caregiver Outcomes: A Longitudinal Study of Irish Spousal Dementia Caregivers. Front Psychol, 10, 11. https://doi.org/10.3389/fpsyg.2019.01801
159. Petriwskyj, A., Parker, D., O'Dwyer, S., Moyle, W., & Nucifora, N. (2016). Interventions to build resilience in family caregivers of people living with dementia: a comprehensive systematic review. JBI Database System Rev Implement Rep, 14(6), 238-273. https://doi.org/10.11124/jbisrir-2016-002555
160. Pfeiffer, K., Theurer, C., Buchele, G., Babac, A., Dick, H., Wilz, G., & ReDiCare Study, G. (2021). Relieving distressed caregivers (ReDiCare study): study protocol of a randomized pragmatic trial. BMC Geriatr, 21(1), 16. https://doi.org/10.1186/s12877-020-01941-w
161. Picot, S. J. F. (1991). The relationship between the rewards, costs, and coping strategies of Black family caregivers. (PH.D.), University of Maryland At Baltimore. Retrieved from https://potsdam.idm.oclc.org/login?url=https://search.ebscohost.com/login.aspx?direct=true&db=rzh&AN=109870462&site=ehost-live&scope=site Available from EBSCOhost CINAHL Plus with Full Text database.
162. Piersol, C. V., Canton, K., Connor, S. E., Giller, I., Lipman, S., & Sager, S. (2017). Effectiveness of Interventions for Caregivers of People With Alzheimer's Disease and. Related Major Neurocognitive Disorders: A Systematic Review. American Journal of Occupational Therapy, 71(5), 10. https://doi.org/10.5014/ajot.2017.027581
163. Pione, R. D., Spector, A., Cartwright, A. V., & Stoner, C. R. (2021). A psychometric appraisal of positive psychology outcome measures in use with carers of people living with dementia: a systematic review. Int Psychogeriatr, 33(4), 385-404. https://doi.org/10.1017/s1041610220003464
164. Plata, T.-L. A. (2007). Stress, coping and meaning in caregivers of family members with Alzheimer disease. (67), ProQuest Information & Learning. Retrieved from https://search.ebscohost.com/login.aspx?direct=true&db=psyh&AN=2007-99008-104&lang=zh-cn&site=ehost-live Available from EBSCOhost APA PsycInfo database.
165. Pleasant, M., Molinari, V., Dobbs, D., Meng, H., & Hyer, K. (2020). Effectiveness of online dementia caregivers training programs: A systematic review. Geriatric Nursing, 41(6), 921-935. https://doi.org/10.1016/j.gerinurse.2020.07.004
166. Polenick, C. A., Sherman, C. W., Birditt, K. S., Zarit, S. H., & Kales, H. C. (2019). Purpose in Life Among Family Care Partners Managing Dementia: Links to Caregiving Gains. Gerontologist, 59(5), E424-E432. https://doi.org/10.1093/geront/gny063
167. Quinn, C., Clare, L., McGuinness, T., & Woods, R. T. (2012). The impact of relationships, motivations, and meanings on dementia caregiving outcomes. Int Psychogeriatr, 24(11), 1816-1826. https://doi.org/10.1017/s1041610212000889
168. Quinn, C., Clare, L., & Woods, R. T. (2012). What predicts whether caregivers of people with dementia find meaning in their role? Int J Geriatr Psychiatry, 27(11), 1195-1202. https://doi.org/10.1002/gps.3773
169. Quinn, C., Nelis, S. M., Martyr, A., Victor, C., Morris, R. G., & Clare, L. (2019). Influence of Positive and Negative Dimensions of Dementia Caregiving on Caregiver Well-Being and Satisfaction With Life: Findings From the IDEAL Study. Am J Geriatr Psychiatry, 27(8), 838-848. https://doi.org/10.1016/j.jagp.2019.02.005
170. Quinn, C., & Toms, G. (2019). Influence of Positive Aspects of Dementia Caregiving on Caregivers' Well-Being: A Systematic Review. Gerontologist, 59(5), E584-E596. https://doi.org/10.1093/geront/gny168
171. Rapp, S. R., & Chao, D. (2000). Appraisals of strain and of gain: effects on psychological wellbeing of caregivers of dementia patients. Aging Ment Health, 4(2), 142-147. https://doi.org/10.1080/13607860050008664
172. Rapp, S. R., Shumaker, S., Schmidt, S., Naughton, M., & Anderson, R. (1998). Social resourcefulness: its relationship to social support and wellbeing among caregivers of dementia victims. Aging Ment Health, 2(1), 40-48. https://doi.org/10.1080/13607869856920
173. Reis, M. F., Gold, D. P., Andres, D., Markiewicz, D., & Gauthier, S. (1994). Personality traits as determinants of burden and health complaints in caregiving. Int J Aging Hum Dev, 39(3), 257-271. https://doi.org/10.2190/6lyn-yfwq-p87d-mkwx
174. Ribeiro, O., Brandão, D., Oliveira, A. F., Teixeira, L., & Paúl, C. (2020). Positive aspects of care in informal caregivers of community‐dwelling dementia patients. Journal of Psychiatric & Mental Health Nursing (John Wiley & Sons, Inc.), 27(4), 330-341. https://doi.org/10.1111/jpm.12582
175. Richards, A. (2020). Finding meaning in caregiving, well-being, and spousal caregivers of people with dementia. (81), ProQuest Information & Learning. Retrieved from https://search.ebscohost.com/login.aspx?direct=true&db=psyh&AN=2020-04050-117&lang=zh-cn&site=ehost-live Available from EBSCOhost APA PsycInfo database.
176. Riedijk, S., Duivenvoorden, H., Van Swieten, J., Niermeijer, M., & Tibben, A. (2009). Sense of Competence in a Dutch Sample of Informal Caregivers of Frontotemporal Dementia Patients. Dement Geriatr Cogn Disord, 27(4), 337-343. https://doi.org/10.1159/000207447
177. Roberts, E., & Struckmeyer, K. M. (2018). The Impact of Respite Programming on Caregiver Resilience in Dementia Care: A Qualitative Examination of Family Caregiver Perspectives. Inquiry-the Journal of Health Care Organization Provision and Financing, 55, 11. https://doi.org/10.1177/0046958017751507
178. Robertson, S. M., Zarit, S. H., Duncan, L. G., Rovine, M. J., & Femia, E. E. (2007). Family Caregivers' Patterns of Positive and Negative Affect. Family Relations: An Interdisciplinary Journal of Applied Family Studies, 56(1), 12-23. https://doi.org/10.1111/j.1741-3729.2007.00436.x
179. Roff, L. L., Burgio, L. D., Gitlin, L., Nichols, L., Chaplin, W., & Hardin, J. M. (2004). Positive aspects of Alzheimer's caregiving: The role of race. Journals of Gerontology Series B-Psychological Sciences and Social Sciences, 59(4), P185-P190. https://doi.org/10.1093/geronb/59.4.P185
180. Roth, D. L., Dilworth-Anderson, P., Huang, J., Gross, A. L., & Gitlin, L. N. (2015). Positive Aspects of Family Caregiving for Dementia: Differential Item Functioning by Race. Journals of Gerontology Series B-Psychological Sciences and Social Sciences, 70(6), 813-819. https://doi.org/10.1093/geronb/gbv034
181. Roud, H., Keeling, S., & Sainsbury, R. (2006). Using the COPE assessment tool with informal carers of people with dementia in New Zealand. N Z Med J, 119(1237), U2053.
182. Rubinstein, R. L. (1989). Themes in the meaning of caregiving. J Aging Stud, 3(2), 119-138. https://doi.org/10.1016/0890-4065(89)90012-1
183. Samia, L. W., O'Sullivan, A., Fallon, K. C., Aboueissa, A., & Hepburn, K. W. (2019). Building on Self-efficacy for Experienced Family Caregivers: The Savvy Advanced Program. Gerontologist, 59(5), 973-982. https://doi.org/10.1093/geront/gny016
184. Sanchez-Teruel, D., Robles-Bello, M. A., Sarhani-Robles, M., & Sarhani-Robles, A. (2022). Exploring resilience and well-being of family caregivers of people with dementia exposed to mandatory social isolation by COVID-19. Dementia-International Journal of Social Research and Practice, 21(2), 410-425. https://doi.org/10.1177/14713012211042187
185. Sanders, S. (2005). Is the glass half empty or full? Reflections on strain and gain in cargivers of individuals with Alzheimer's disease. Soc Work Health Care, 40(3), 57-73. https://doi.org/10.1300/J010v40n03_04
186. Sarabia-Cobo, C., & Sarria, E. (2021). Satisfaction with caregiving among informal caregivers of elderly people with dementia based on the salutogenic model of health. Applied Nursing Research, 62, 6. https://doi.org/10.1016/j.apnr.2021.151507
187. Savundranayagam, M. Y. (2014). Receiving while giving: The differential roles of receiving help and satisfaction with help on caregiver rewards among spouses and adult‐children. Int J Geriatr Psychiatry, 29(1), 41-48. https://doi.org/10.1002/gps.3967
188. Semiatin, A. M., & O'Connor, M. K. (2012). The relationship between self-efficacy and positive aspects of caregiving in Alzheimer's disease caregivers. Aging Ment Health, 16(6), 683-688. https://doi.org/10.1080/13607863.2011.651437
189. Senturk, S. G., Soylemez, B. A., Akyol, M. A., Isik, A. T., & Kucukguclu, O. (2021). Psychometric properties of the Turkish version of the scale for positive aspects of caregiving experience. Perspect Psychiatr Care, 6. https://doi.org/10.1111/ppc.12973
190. Sheth, K. (2020). Alzheimer's 'the family disease': Examining the effects of resilience on preparedness and compassion in Asian family caregivers. (81), ProQuest Information & Learning. Retrieved from https://search.ebscohost.com/login.aspx?direct=true&db=psyh&AN=2020-28117-169&lang=zh-cn&site=ehost-live Available from EBSCOhost APA PsycInfo database.
191. Shim, B., Barroso, J., & Davis, L. L. (2012). A comparative qualitative analysis of stories of spousal caregivers of people with dementia: Negative, ambivalent, and positive experiences. Int J Nurs Stud, 49(2), 220-229. https://doi.org/10.1016/j.ijnurstu.2011.09.003
192. Shim, B., Barroso, J., Gilliss, C. L., & Davis, L. L. (2013). Finding meaning in caring for a spouse with dementia. Applied Nursing Research, 26(3), 121-126. https://doi.org/10.1016/j.apnr.2013.05.001
193. Shrestha, S., Richey, S., Lipovac-Dew, M., Kunik, M. E., Stanley, M. A., Ramsey, D., & Amspoker, A. B. (2020). An Examination of Positive and Negative Dementia Caregiving Experiences. Clin Gerontol, 10. https://doi.org/10.1080/07317115.2020.1868033
194. Shyu, Y. I. L., Yang, C. T., Huang, C. C., Kuo, H. C., Chen, S. T., & Hsu, W. C. (2010). Influences of Mutuality, Preparedness, and Balance on Caregivers of Patients With Dementia. Journal of Nursing Research, 18(3), 155-163. https://doi.org/10.1097/JNR.0b013e3181ed5845
195. Siow, J. Y. M., Chan, A., Ostbye, T., Cheng, G. H. L., & Malhotra, R. (2017). Validity and Reliability of the Positive Aspects of Caregiving (PAC) Scale and Development of Its Shorter Version (S-PAC) Among Family Caregivers of Older Adults. Gerontologist, 57(4), E75-E84. https://doi.org/10.1093/geront/gnw198
196. Skarupski, K. A., McCann, J. J., Bienias, J. L., & Evans, D. A. (2009). Race differences in emotional adaptation of family caregivers. Aging Ment Health, 13(5), 715-724. https://doi.org/10.1080/13607860902845582
197. Smaling, H. J. A., Joling, K. J., Achterberg, W. P., Francke, A. L., & van der Steen, J. T. (2021). Measuring positive caregiving experiences in family caregivers of nursing home residents: A comparison of the Positive Experiences Scale, Gain in Alzheimer Care INstrument, and Positive Aspects of Caregiving questionnaire. Geriatr Gerontol Int, 21(8), 636-643. https://doi.org/10.1111/ggi.14210
198. Smith, A. L. (1999). The forgotten victim: An in-depth look and an overall assessment of Alzheimer's caregivers' needs, struggles, and satisfactions (alzheimer's disease). (60), ProQuest Information & Learning. Retrieved from https://search.ebscohost.com/login.aspx?direct=true&db=psyh&AN=1999-95019-024&lang=zh-cn&site=ehost-live Available from EBSCOhost APA PsycInfo database.
199. Son, G. R., Wykle, M. L., & Zauszniewski, J. A. (2003). Korean Adult Child Caregivers of Older Adults with Dementia: Predictors of Burden and Satisfaction. J Gerontol Nurs, 29(1), 19-28. https://doi.org/10.3928/0098-9134-20030101-09
200. Stansfeld, J., Crellin, N., Orrell, M., Wenborn, J., Charlesworth, G., & Vernooij-Dassen, M. (2019). Factors related to sense of competence in family caregivers of people living with dementia in the community: a narrative synthesis. Int Psychogeriatr, 31(6), 799-813. https://doi.org/10.1017/s1041610218001394
201. Stansfeld, J., Stoner, C. R., Wenborn, J., Vernooij-Dassen, M., Moniz-Cook, E., & Orrell, M. (2017). Positive psychology outcome measures for family caregivers of people living with dementia: a systematic review. Int Psychogeriatr, 29(8), 1281-1296. https://doi.org/10.1017/s1041610217000655
202. Sterritt, P. F., & Pokorny, M. E. (1998). African-American caregiving for a relative with Alzheimer's disease. Geriatric Nursing, 19(3), 127-+. https://doi.org/10.1016/s0197-4572(98)90056-8
203. Stockwell-Smith, G., Moyle, W., & Kellett, U. (2018). The impact of early psychosocial intervention on self-efficacy of care recipient/carer dyads living with early-stage dementiaA mixed-methods study. J Adv Nurs, 74(9), 2167-2180. https://doi.org/10.1111/jan.13710
204. Talkington-Boyer, S., & Snyder, D. K. (1994). Assessing impact on family caregivers to Alzheimer's disease patients. American Journal of Family Therapy, 22(1), 57-66. https://doi.org/10.1080/01926189408251297
205. Tarlow, B. J., Wisniewski, S. R., Belle, S. H., Rubert, M., Ory, M. G., & Gallagher-Thompson, D. (2004). Positive aspects of caregiving: contributions of the REACH Project to the development of new measures for Alzheimer's caregiving. Res Aging, 26(4), 429-453. https://doi.org/10.1177/0164027504264493
206. Teahan, A., Lafferty, A., McAuliffe, E., Phelan, A., O'Sullivan, L., O'Shea, D., & Fealy, G. (2018). Resilience in family caregiving for people with dementia: A systematic review. Int J Geriatr Psychiatry, 33(12), 1582-1595. https://doi.org/10.1002/gps.4972
207. Teles, S., Ferreira, A., Seeher, K., Freel, S., & Paul, C. (2020). Online training and support program (iSupport) for informal dementia caregivers: protocol for an intervention study in Portugal. BMC Geriatr, 20(1), 13. https://doi.org/10.1186/s12877-019-1364-z
208. Tookey, S. A., Greaves, C. V., Rohrer, J. D., & Stott, J. (2021). Specific support needs and experiences of carers of people with frontotemporal dementia: A systematic review. Dementia-International Journal of Social Research and Practice, 20(8), 3032-3054. https://doi.org/10.1177/14713012211022982
209. Tretteteig, S., Vatne, S., & Rokstad, A. M. M. (2017). Meaning in family caregiving for people with dementia: a narrative study about relationships, values, and motivation, and how day care influences these factors. J Multidiscip Healthc, 10, 445-455. https://doi.org/10.2147/jmdh.S151507
210. Tsatali, M., Egkiazarova, M., Toumpalidou, M., Karagiozi, K., Margaritidou, P., & Tsolaki, M. (2019). Greek Adaptation of the Positive Aspects of Caregiving (PAC) Scale in Dementia Caregivers. Clin Gerontol, 10. https://doi.org/10.1080/07317115.2019.1685047
211. Tulloch, K., McCaul, T., & Scott, T. L. (2022). Positive Aspects of Dementia Caregiving During the COVID-19 Pandemic. Clin Gerontol, 45(1), 86-96. https://doi.org/10.1080/07317115.2021.1929630
212. Uwakwe, R. (2006). Satisfaction with dementia care--giving in Nigeria--a pilot investigation. Int J Geriatr Psychiatry, 21(3), 296-297. https://doi.org/10.1002/gps.1500
213. Vernon-Scott, S. A. (2008). Positive and negative caregiver appraisal and caregiver health outcomes. (68), ProQuest Information & Learning. Retrieved from https://search.ebscohost.com/login.aspx?direct=true&db=psyh&AN=2008-99080-593&lang=zh-cn&site=ehost-live Available from EBSCOhost APA PsycInfo database.
214. Vidigal, F. C., Rogonni Ferrari, R. F., Murio Ribeiro Rodrigues, D. M., Silva Marcon, S., Denardi Antoniassi Baldissera, V., & Carreira, L. (2014). Satisfaction in caring for older adults with alzheimer's: perceptions of the family caregivers. Cogitare Enfermagem, 19(4), 708-715.
215. Wai Kit, T. (2017). Psychosocial Intervention for Dementia Caregiver on Enhancing Self-efficacy. Psychosocial Intervention for Dementia Caregiver on Enhancing Self-Efficacy, 1-1.
216. Walmsley, B. D., & McCormack, L. (2016). Synthesis of Meaning: Negative and Positive Change in Family Members Following the Adversity of Dementia. Journal of Humanistic Psychology, 56(2), 122-143. https://doi.org/10.1177/0022167814557547
217. Wang, Z. X., Ma, C. Y., Han, H. J., He, R. L., Zhou, L. Y., Liang, R. F., & Yu, H. M. (2018). Caregiver burden in Alzheimer's disease: Moderation effects of social support and mediation effects of positive aspects of caregiving. Int J Geriatr Psychiatry, 33(9), 1198-1206. https://doi.org/10.1002/gps.4910
218. Wawrziczny, E., Antoine, P., & Doba, K. (2021). Modeling the Distress of Adult-Child Caregivers of People with Dementia: The Mediating Role of Self-Efficacy. Journal of Alzheimers Disease, 84(2), 855-867. https://doi.org/10.3233/jad-210624
219. Whelan, S., Teahan, A., & Casey, D. (2020). Fostering the Resilience of People With Dementia: A Narrative Literature Review. Front Med (Lausanne), 7, 15. https://doi.org/10.3389/fmed.2020.00045
220. Wilks, S. E., & Croom, B. (2008). Perceived stress and resilience in Alzheimer's disease caregivers: testing moderation and mediation models of social support. Aging Ment Health, 12(3), 357-365. https://doi.org/10.1080/13607860801933323
221. Williamson, T., & Paslawski, T. (2016). Resilience in dementia: Perspectives of those living with dementia. Canadian Journal of Speech-Language Pathology and Audiology, 40(1), 1-15.
222. Wu, Q., Yamaguchi, Y., & Greiner, C. (2022). Factors associated with the well‐being of family caregivers of people with dementia. Psychogeriatrics. https://doi.org/10.1111/psyg.12805
223. Wylie, M. J., Kim, K., Liu, Y., & Zarit, S. H. (2021). Taking a Break: Daily Respite Effects of Adult Day Services as Objective and Subjective Time Away From Caregiving. Gerontologist, 61(8), 1231-1240. https://doi.org/10.1093/geront/gnaa178
224. Xue, H. H., Zhai, J. W., He, R. L., Zhou, L. Y., Liang, R. F., & Yu, H. M. (2018). Moderating role of positive aspects of caregiving in the relationship between depression in persons with Alzheimer's disease and caregiver burden. Psychiatry Res, 261, 400-405. https://doi.org/10.1016/j.psychres.2017.12.088
225. Yap, P., Luo, N., Ng, W. Y., Chionh, H. L., Lim, J., & Goh, J. (2010). Gain in Alzheimer Care INstrument-A New Scale to Measure Caregiving Gains in Dementia. American Journal of Geriatric Psychiatry, 18(1), 68-76. <https://doi.org/10.1097/JGP.0b013e3181bd1dcd>
226. Yu, D. S. F., Cheng, S. T., & Kwok, T. (2021). Developing and testing of an integrative theoretical model to predict positive aspects of caregiving among family caregivers of persons with dementia: A study protocol. J Adv Nurs, 77(1), 401-410. https://doi.org/10.1111/jan.14561
227. Yu, D. S. F., Cheng, S. T., & Wang, J. F. (2018). Unravelling positive aspects of caregiving in dementia: An integrative review of research literature. Int J Nurs Stud, 79, 1-26. https://doi.org/10.1016/j.ijnurstu.2017.10.008
228. Yu, H., Wu, L., Chen, S., Wu, Q., Yang, Y., & Edwards, H. (2016). Caregiving burden and gain among adult-child caregivers caring for parents with dementia in China: the partial mediating role of reciprocal filial piety. Int Psychogeriatr, 28(11), 1845-1855. https://doi.org/10.1017/s1041610216000685
229. Yu, H. M., Wang, X. C., He, R. L., Liang, R. F., & Zhou, L. Y. (2015). Measuring the Caregiver Burden of Caring for Community-Residing People with Alzheimer's Disease. PLoS One, 10(7), 13. https://doi.org/10.1371/journal.pone.0132168
230. Zhang, S. Y., Edwards, H., Yates, P., Guo, Q. H., & Li, C. B. (2013). Partial Mediation Role of Self-Efficacy between Positive Social Interaction and Mental Health in Family Caregivers for Dementia Patients in Shanghai. PLoS One, 8(12), 7. <https://doi.org/10.1371/journal.pone.0083326>

**Supplementary S3: Forms of positive aspects of caregiving and related measurements**

**1. Positive aspects of caregiving**

The Positive Aspects of Caregiving Scale (Tarlow et al., 2004) #

The Positive and Negative Affect Scale (PANAS) (Watson, Clark, & Tellegen, 1988)

The Scale for Positive Aspects of Caregiving Experience (SPACE)-44 (Kate, 2012)

Dementia Caregiver Positive Feelings Scale 21-item version (DCPFS-21) (Fuju, Yamagami, Yamaguchi, & Yamazaki, 2021)

**2. Satisfaction**

Satisfaction with Life Scale (SWLS) (Diener, 1985)

Participant satisfaction (Woods, 1984)

Caregiver satisfaction scale (Lawton, 1989)

Satisfaction with caregiving role (George, 1986)

The 24-item Marital Needs Satisfaction Scale (Stinnett, 1970)

**3.Self-efficacy**

Caregiving self-efficacy scale (CSES-15) (Au, 2009)

Self-efficacy questionnaire (Fortinsky, 2002)

Self-Efficacy Scale (Kuhn, 2004)

Coping Self- Efficacy Scale (Chesney, 2006)

Revised Scale for Caregiving self- efficacy (Steffen, 2002) #

General Self-efficacy Scale (GS-ES) (Schwarzer R., 1995)

**4. well-being**

A self-developed visual analogue scale of well-being (Wilz, Meichsner, & Soellner, 2017)

The Dupuy Psychological General Weil-Being Index (Dupuy, 1984)

The Caregiver Well-Being Scale (Tebb, 1995)

The Psychological General Well-Being Inventory (PGWBI) (Grossi, 2006)

The 18-item version of Ryff's Psychological Well-Being Scale (Ryff, 1989)

**5.** **Gains**

Caregiver Personal Gain Scale(4-item) (Pearlin, Mullan, Semple, & Skaff, 1990)

Appraisals of gain——11 items (Rapp, 2000)

Gain in Alzheimer's care Instrument (GAIN)(Yap, 2010) #

The Gains Associated with Caregiving (GAC) scale (Faba, 2017)

A scale contains 4 items (Polenick, 2019)

**6.** **Competence**

Caregiving Competence scale (Pearlin, Mullan, Semple, & Skaff, 1990)

The Sense of Competence Questionnaire (SCQ)-27 (Scholte op Reimer, 1998)

The Sense of Competence Questionnaire (SCQ)-28 (Vernooij-Dassen, 1996)

Sense of Competence Questionnaire (SCQ-AV) (Pendergrass, 2015)

**7.Resilience**

Connor-Davidson Resilience Scale (CD-RISC) (Connor, 2003)

Brief Resilient Coping Scale (BRCS) (Sinclair, 2004)

The Family Sense of Coherence (FSC) Questionnaire (meaning) (Antonovsky, 1988)

Positive Psychology Outcome Measure (PPOM)-16 (Stoner, 2018)

The Resilience Scale (Pesce, 2005)

**8.Meaning**

The 12-item Meaning in Caregiving Scale (Noonan, 1997)

The Finding Meaning Through Caregiving Scale (Farran, 1997) #

**9.Hope**

The Miller Hope Scale (Miller & Powers, 1988)

Herth Hope Index (HHI-12) (Herth, 1992)

**10.Rewards**

Caregiver rewards scale—24 items (Picot, 1991)

The three subscales of the FCI Rewards of Caregiving Scale (Archbold, 1992)

**11. Other variables of positive aspects of caregiving**

Caregiver Mastery Scale（7-item）(Pearlin et al., 1990)

Self-rated health (HEALTH)-11(Stewart, 1992)

The COPE Index (McKee, 2003)

The Mutuality scale (Archbold, 1992)

The 28-item Resourcefulness Scale (RS) (Zauszniewski, 2006)

Life Orientation Test Revised (LOT-R) (Scheier, 1994)

Caregiver Reaction Assessment (CRA) questionnaire (24 items in five subscales) (Given, 1992)

The Positive Thinking Skills Scale (PTSS) (Bekhet, 2013)

The Caregiving Gratification Scale (Nishimura, Suda, & Campbell, 2005)

The positive affect subscale of the Bradburn Affect Balance Scale (Bradburn, 1969)

Positive affect (PA) scale-10 items (Watson et al., 1988)

Caregiving Hassles and Uplifts Scale (Kinney, 1987)

Ryff (1989)general personal growth scale (Ryff, 1989)

Positive views toward caregiving-3 (Boise, 2005)

*# Most cited in the study and provided detailed description in the manscript (Table 2)*

**References:**

Antonovsky, A., & Sourani, T. (1988). Family sense of coherence and family adaptation. *Journal of Marriage and the Family*, 79-92.

Archbold, P. G. (1992). The clinical assessment of mutuality and preparedness in family caregiver to frail older poaple. *Key aspects of elder care*, 328-339.

Au, A., Lai, M. K., Lau, K. M., Pan, P. C., Lam, L., Thompson, L., & Gallagher-Thompson, D. (2009). Social support and well-being in dementia family caregivers: the mediating role of self-efficacy. *Aging & mental health, 13*, (5), 761–768. https://doi.org/https://doi.org/10.1080/13607860902918223

Bekhet, A. K. (2013). Effects of positive cognitions and resourcefulness on caregiver burden among caregivers of persons with dementia. *International Journal of Mental Health Nursing, 22(4)*, 340-346. https://doi.org/https://doi.org/10.1111/j.1447-0349.2012.00877.x

Boise, L., Congleton, L., & Shannon, K. (2005). Empowering family caregivers: The powerful tools for caregiving program. *Educational Gerontology, 31(7)*, 573-586.

Bradburn, N. M. (1969). The structure of psychological well-being.

Chesney, M. A., Neilands, T. B., Chambers, D. B., Taylor, J. M., & Folkman, S. (2006). A validity and reliability study of the coping self-efficacy scale. *British journal of health psychology*, 11(Pt 13), 421–437. https://doi.org/https://doi.org/10.1348/135910705X53155

Connor, K. M., & Davidson, J. R. (2003). Development of a new resilience scale: The Connor‐Davidson resilience scale (CD‐RISC). *Depression and anxiety, 18(2)*, 76-82.

Diener, E., Emmons, R. A., Larsen, R. J., & Griffin, S. (1985). The Satisfaction With Life Scale. *Journal of personality assessment, 49(1)*, 71–75. https://doi.org/https://doi.org/10.1207/s15327752jpa4901_13

Dupuy, H. J. (1984). The psychological general well-being (PGWB) index. *Assessment of quality of life in clinical trials of cardiovascular therapies*, 170-183.

Faba, J., Villar, F., & Giuliani, M. F. (2017). Development of a measure to evaluate gains among spanish dementia caregivers: The gains associated with caregiving (GAC) scale. *Arch Gerontol Geriatr, 68*, 76-83. https://doi.org/https://doi.org/10.1016/j.archger.2016.09.004

Farran, C. J. (1997). Theoretical perspectives concerning positive aspects of caring for elderly persons with dementia: stress/adaptation with existentialism. *37(2)*, 250-256. https://doi.org/https://doi.org/10.1093/geront/37.2.250

Fortinsky, R. H., Kercher, K., & Burant, C. J. (2002). Measurement and correlates of family caregiver self-efficacy for managing dementia. *Aging & mental health, 6*, (2), 153–160. https://doi.org/ https://doi.org/10.1080/13607860220126763

Fuju, T., Yamagami, T., Yamaguchi, H., & Yamazaki, T. (2021). Development of the Dementia Caregiver Positive Feeling Scale 21‐item version (DCPFS‐21) in Japan to recognise positive feelings about caregiving for people with dementia. *Psychogeriatrics, 21*(4), 650-658. https://doi.org/10.1111/psyg.12727

George, L. K., & Gwyther, L. P. (1986). Caregiver well-being: a multidimensional examination of family caregivers of demented adults. *The Gerontologist, 26(3)*, 253–259. https://doi.org/ https://doi.org/10.1093/geront/26.3.253

Given, C. W., Given, B., Stommel, M., Collins, C., King, S., & Franklin, S. (1992). The caregiver reaction assessment (CRA) for caregivers to persons with chronic physical and mental impairments. *Research in nursing & health, 15(4)*, 271–283. https://doi.org/ https://doi.org/10.1002/nur.4770150406

Grossi, E., Groth, N., Mosconi, P., Cerutti, R., Pace, F., Compare, A., & Apolone, G. (2006). Development and validation of the short version of the Psychological General Well-Being Index (PGWB-S). *Health and quality of life outcomes, 4(1)*, 1-8.

Herth, K. (1992). Abbreviated instrument to measure hope: development and psychometric evaluation. *Journal of advanced nursing, 17(10)*, 1251-1259.

Kate, N., Grover, S., Kulhara, P., & Nehra, R. . (2012). Scale for positive aspects of caregiving experience: development, reliability, and factor structure. *East Asian Archives of Psychiatry*, 22(22), 62-69.

Kinney, J. M., & Stephens, M. A. P. (1987). The caregiving hassles scale: Administration, reliability and validity. *Kent, OH: Kent State University, Psychology Department*.

Kuhn, D., & Fulton, B. R. (2004). Efficacy of an educational program for relatives of persons in the early stages of Alzheimer's disease. *Journal of Gerontological Social Work, 42*, (3-4), 109-130.

Lawton, M. P., Kleban, M. H., Moss, M., Rovine, M., & Glicksman, A. (1989). Measuring caregiving appraisal. J Gerontol, 44(3), P61-71. https://doi.org/10.1093/geronj/44.3.p61

McKee, K. J., Philp, I., Lamura, G., Prouskas, C., Oberg, B., Krevers, B., Spazzafumo, L., Bień, B., Parker, C., Nolan, M. R., Szczerbinska, K., & COPE Partnership. (2003). The COPE index--a first stage assessment of negative impact, positive value and quality of support of caregiving in informal carers of older people. *Aging & mental health, 7(1)*, 39–52. https://doi.org/ https://doi.org/10.1080/1360786021000006956

Miller, J. F., & Powers, M. J. (1988). Development of an instrument to measure hope. *Nurs Res, 37*(1), 6-10.

Nishimura, M., Suda, R., & Campbell, R. (2005). Scale development of Caregiving Gratification Scale: Positive appraisal assessment in family caregivers care experience. *Kousei no Shihyo,, 52*, 8-13.

Noonan, A. E., & Tennstedt, S. L. (1997). Meaning in caregiving and its contribution to caregiver well-being. *Gerontologist, 37(6)*, 785-794. https://doi.org/ https://doi.org/10.1093/geront/37.6.785

Pearlin, L. I., Mullan, J. T., Semple, S. J., & Skaff, M. M. (1990). Caregiving and the stress process: an overview of concepts and their measures. *Gerontologist, 30*(5), 583-594. https://doi.org/10.1093/geront/30.5.583

Pendergrass, A., Beische, D., Becker, C., Hautzinger, M., & Pfeiffer, K. . (2015). An abbreviated German version of the Sense of Competence Questionnaire among informal caregivers of relatives who had a stroke: development and validation. *European journal of ageing*, 12(13), 203–213. https://doi.org/https://doi.org/10.1007/s10433-015-0342-3

Pesce, R. P., Assis, S. G., Avanci, J. Q., Santos, N. C., Malaquias, J. V., & Carvalhaes, R. (2005). Cross-cultural adaptation, reliability and validity of the resilience scale. *Cadernos de saude publica,  21(2)*, 436-448.

Picot, S. J. F. (1991). The relationship between the rewards, costs, and coping strategies of Black family caregivers. (PH.D.), University of Maryland At Baltimore. Retrieved from https://potsdam.idm.oclc.org/login?url=https://search.ebscohost.com/login.aspx?direct=true&db=rzh&AN=109870462&site=ehost-live&scope=site Available from EBSCOhost CINAHL Plus with Full Text database.

Polenick, C. A., Sherman, C. W., Birditt, K. S., Zarit, S. H., & Kales, H. C. (2019). Purpose in Life Among Family Care Partners Managing Dementia: Links to Caregiving Gains. *Gerontologist, 59(5)*, E424-E432. https://doi.org/https://doi.org/10.1093/geront/gny063

Rapp, S. R., & Chao, D. (2000). Appraisals of strain and of gain: effects on psychological wellbeing of caregivers of dementia patients. *Aging & Mental Health, 4(2)*, 142-147. https://doi.org/https://doi.org/10.1080/13607860050008664

Ryff, C. D. (1989). Happiness is everything, or is it? Explorations on the meaning of psychological well-being. *Journal of personality and social psychology, 57(6)*, 1069-1081.

Scheier, M. F., Carver, C. S., & Bridges, M. W. (1994). Distinguishing optimism from neuroticism (and trait anxiety, self-mastery, and self-esteem): a reevaluation of the Life Orientation Test. *Journal of personality and social psychology, 67(6)*, 1063–1078. https://doi.org/ https://doi.org/10.1037//0022-3514.67.6.1063

Scholte op Reimer, W. J., de Haan, R. J., Pijnenborg, J. M., Limburg, M., & van den Bos, G. A. ( 1998). Assessment of burden in partners of stroke patients with the sense of competence questionnaire. (Stroke), 29(22), 373–379. https://doi.org/ https://doi.org/10.1161/01.str.29.2.373

Schwarzer R., J. M. (1995). Spanish adaptation of the general self-efficacy scale. *Measures in health psychology: A user’s portfolio. Causal and control beliefs, NFER-NELSON*.

Sinclair, V. G., & Wallston, K. A. (2004). The development and psychometric evaluation of the Brief Resilient Coping Scale. *Assessment, 11(1)*, 94-101.

Steffen, A. M., McKibbin, C., Zeiss, A. M., Gallagher-Thompson, D., & Bandura, A. (2002). The revised scale for caregiving self-efficacy: reliability and validity studies. *The journals of gerontology. Series B, Psychological sciences and social sciences, 57*, (1), P74–P86. https://doi.org/https://doi.org/10.1093/geronb/57.1.p74

Stewart, A. L. (1992). Measuring functioning and well-being: the medical outcomes study approach. *duke university Press*.

Stinnett, N., Collins, J., & Montgomery, J. E. (1970). Marital need satisfaction of older husbands and wives. *Journal of Marriage and the Family*, 428-434.

Stoner, C. R., Orrell, M., & Spector, A. (2018). The Positive Psychology Outcome Measure (PPOM) for people with dementia: psychometric properties and factor structure. *Archives of gerontology and geriatrics, 76*, 182-187.

Tarlow, B. J., Wisniewski, S. R., Belle, S. H., Rubert, M., Ory, M. G., & Gallagher-Thompson, D. (2004). Positive aspects of caregiving: contributions of the REACH Project to the development of new measures for Alzheimer's caregiving. *Res Aging, 26*(4), 429-453. https://doi.org/10.1177/0164027504264493

Tebb, S. (1995). An aid to empowerment: a caregiver well-being scale. *Health & social work, 20(2)*, 87–92. https://doi.org/ https://doi.org/10.1093/hsw/20.2.87

Vernooij-Dassen, M. J., Persoon, J. M., & Felling, A. J. . (1996). Predictors of sense of competence in caregivers of demented persons. *Social science & medicine (1982)*, 43(41), 41–49. https://doi.org/https://doi.org/10.1016/0277-9536(95)00332-0

Watson, D., Clark, L. A., & Tellegen, A. (1988). Development and validation of brief measures of positive and negative affect: the PANAS scales. *J Pers Soc Psychol, 54*(6), 1063-1070. https://doi.org/10.1037//0022-3514.54.6.1063

Wilz, G., Meichsner, F., & Soellner, R. (2017). Are psychotherapeutic effects on family caregivers of people with dementia sustainable? Two-year long-term effects of a telephone-based cognitive behavioral intervention. *Aging Ment Health, 21*(7), 774-781. https://doi.org/10.1080/13607863.2016.1156646

Woods, A. M. (1984). Support groups; Who joins them and do they deliver? *In Paper presented at the meetings of the American Psychological Association, Toronto, Ontario, Canada, 1984*.

Yap, P., Luo, N., Ng, W. Y., Chionh, H. L., Lim, J., & Goh, J. (2010). Gain in Alzheimer Care INstrument-A New Scale to Measure Caregiving Gains in Dementia. American Journal of Geriatric Psychiatry. *18(1)*, 68-76. https://doi.org/https://doi.org/10.1097/JGP.0b013e3181bd1dcd

Zauszniewski, J. A., Lai, C. Y., & Tithiphontumrong, S. (2006). Development and testing of the resourcefulness scale for older adults. *Journal of Nursing Measurement, 14(1)*, 57-68.

**Supplementary S4:** **Theories most cited in the studies**

| **Theories** | **N** | **Description** | **Elements** |
| --- | --- | --- | --- |
| Lazarus' stress model (Lazarus & Folkman, 1986) | 15 | A book named "stress, appraisal, and coping" extensively articulates a theory of psychological stress based on cognitive evaluation and coping. | Stress concept, cognitive appraisal process, person and situation influencing factors, coping process, outcomes, etc. |
| Stress process model (Pearlin, Mullan, Semple, & Skaff, 1990) | 11 | It views caregiver stress as a consequence of a process comprising a number of interrelated conditions, including the socioeconomic characteristics and resources of caregivers and the primary and secondary stressors to which they are exposed | background/contextual variables, Primary stressors, Secondary stressors( Role strains and intrapsychic strains), Mediating Conditions (social support, coping), outcomes |
| Revised Stress and coping theory (Folkman, 1997) | 3 | It describes coping processes that are associated with positive psychological states in the context of intense distress and discusses the theoretical implications of positive psychological states in the coping process | event, appraisal, coping, event outcome, emotion outcome |
| Noonan and Tennstedt model (Noonan & Tennstedt, 1997) | 2 | It provides a useful model for incorporating the construct of meaning in caregiving into the stress process framework (Pearlin, et al, 1990) used to study caregiving | background/contextual variables, stressors, mediators, outcomes. Incorporating meaning in mediators. |
| Stress-buffering model (Cobb, 1976) | 1 | This model proposes that support is related to well-being and protects, or “buffers” individuals from the potentially pathogenic influence of stressful events | social support, life stress |
| The adaptive stress-appraisal model of Chappell and Reid (Chappell & Reid, 2002) | 1 | It is adapted from Yates et al (1999) model, built on the stress process model of Pearlin (1990), to further explore the relationship between burden and well-being. The finding that perceived social support is strongly related to well-being but unrelated to burden reinforces the conceptual distinctiveness of the latter two concepts. | Primary stressors, primary appraisal, mediators, secondary appraisal, outcome |
| Resilience theory (Luthar, Cicchetti, & Becker, 2000) | 5 | It presents a critical appraisal of resilience, a construct connoting the maintenance of positive adaptation by individuals despite experiences of significant adversity. This theory proposes that resilience manifests the interplay between risk factors and protective factors in the face of hardship | Not applicable |
| The conceptual model of dementia caregiver resilience of Joling et al (2016) | 1 | It was based on the theoretical resilience framework of Windle and Bennett (2011) and the research of Gaugler et al (2007). resilience can be described as ‘the process of negotiating, managing and adapting to significant sources of stress or trauma. Assets and resources within the individual, their life and environment facilitate this capacity for adaptation and “bouncing back” in the face of adversity’ | Demands, resilience, Resources (individual, community, and social resources, context of care), consequence |
| Resilience framework (Windle, 2012) | 1 | It identifies general pathways that can guide the promotion of resilience through reducing the threat/adversity and promoting the development of assets and resources to facilitate a good outcome | Antecedents, risk or resilience (presence or absence of resources), consequences |
| Positive psychology theory (Windle, 2012) | 4 | The positive psychology framework uses the study of strengths, virtues, and positive emotions that enable people to thrive (Seligman et al., 2005) to achieve a greater understanding of well-being, even in the face of difficult circumstances | Not applicable |

**References:**

Chappell, N. L., & Reid, R. C. (2002). Burden and well-being among caregivers: examining the distinction. *Gerontologist, 42*(6), 772-780. https://doi.org/10.1093/geront/42.6.772

Cobb, S. (1976). Presidential Address-1976. Social support as a moderator of life stress. *Psychosom Med, 38*(5), 300-314. https://doi.org/10.1097/00006842-197609000-00003

Folkman, S. (1997). Positive psychological states and coping with severe stress. *Soc Sci Med, 45*(8), 1207-1221. https://doi.org/10.1016/s0277-9536(97)00040-3

Gaugler, J. E., Kane, R. L., & Newcomer, R. (2007). Resilience and transitions from dementia caregiving. *J Gerontol B Psychol Sci Soc Sci, 62*(1), P38-44. https://doi.org/10.1093/geronb/62.1.p38

Joling, K. J., Windle, G., Dröes, R. M., Meiland, F., van Hout, H. P., MacNeil Vroomen, J., . . . Woods, B. (2016). Factors of Resilience in Informal Caregivers of People with Dementia from Integrative International Data Analysis. *Dement Geriatr Cogn Disord, 42*(3-4), 198-214. https://doi.org/10.1159/000449131

Lazarus, R. S., & Folkman, S. (1986). *Stress, appraisal, and coping*. New York: Springer.

Luthar, S. S., Cicchetti, D., & Becker, B. (2000). The construct of resilience: a critical evaluation and guidelines for future work. *Child Dev, 71*(3), 543-562. https://doi.org/10.1111/1467-8624.00164

Noonan, A. E., & Tennstedt, S. L. (1997). Meaning in caregiving and its contribution to caregiver well-being. *Gerontologist, 37*(6), 785-794. https://doi.org/10.1093/geront/37.6.785

Pearlin, L. I., Mullan, J. T., Semple, S. J., & Skaff, M. M. (1990). Caregiving and the stress process: an overview of concepts and their measures. *Gerontologist, 30*(5), 583-594. https://doi.org/10.1093/geront/30.5.583

Windle, G. (2012). The contribution of resilience to healthy ageing. *Perspect Public Health, 132*(4), 159-160. https://doi.org/10.1177/1757913912449572

Yates, M. E., Tennstedt, S., & Chang, B. H. (1999). Contributors to and mediators of psychological well-being for informal caregivers. *J Gerontol B Psychol Sci Soc Sci, 54*(1), P12-22. https://doi.org/10.1093/geronb/54b.1.p12
